# Accurate spike estimation from noisy calcium signals for ultrafast three-dimensional imaging of large neuronal populations *in vivo*

**DOI:** 10.1038/ncomms12190

**Published:** 2016-07-19

**Authors:** Thomas Deneux, Attila Kaszas, Gergely Szalay, Gergely Katona, Tamás Lakner, Amiram Grinvald, Balázs Rózsa, Ivo Vanzetta

**Affiliations:** 1 Institut de Neurosciences de la Timone (INT), CNRS and Aix-Marseille Université, UMR 7289, 27 boulevard Jean Moulin, Marseille 13005, France; 2 CNRS FRE-3693, Unité de Neurosciences Information et Complexité, 1 Avenue de la Terrasse, Gif-sur-Yvette 91198, France; 3 Aix Marseille Université, Institut de Neuroscience des Systèmes, Marseille 13005, France; 4 Inserm, UMR_S 1106, 27 Bd Jean Moulin, Marseille Cedex 5 13385, France; 5 Two-Photon Imaging Center, Institute of Experimental Medicine, Hungarian Academy of Sciences, Budapest 1083, Hungary; 6 Faculty of Information Technology and Bionics, Pázmány Péter Catholic University, Budapest 1083, Hungary; 7 Neurobiology Department, Weizmann Institute of Science, Rehovot 76100, Israel

## Abstract

Extracting neuronal spiking activity from large-scale two-photon recordings remains challenging, especially in mammals *in vivo*, where large noises often contaminate the signals. We propose a method, MLspike, which returns the most likely spike train underlying the measured calcium fluorescence. It relies on a physiological model including baseline fluctuations and distinct nonlinearities for synthetic and genetically encoded indicators. Model parameters can be either provided by the user or estimated from the data themselves. MLspike is computationally efficient thanks to its original discretization of probability representations; moreover, it can also return spike probabilities or samples. Benchmarked on extensive simulations and real data from seven different preparations, it outperformed state-of-the-art algorithms. Combined with the finding obtained from systematic data investigation (noise level, spiking rate and so on) that photonic noise is not necessarily the main limiting factor, our method allows spike extraction from large-scale recordings, as demonstrated on acousto-optical three-dimensional recordings of over 1,000 neurons *in vivo*.

## Abstract (web summary)

Two-photon laser scanning microscopy allows functional calcium imaging of large neuronal populations *in vivo*, but the recorded signals typically suffer from low signal to noise. Here the authors develop an algorithm, MLspike, which estimates action potentials from noisy calcium signals, and benchmark it against existing methods.

## Graphical abstract

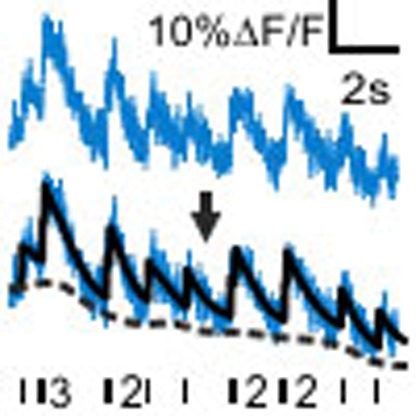

To understand how local networks process information, we need experimental access to the activity of large sets of individual neurons *in vivo*. Unlike multi-electrode probes^1^,^2^,^3^, two-photon laser scanning microscopy^4^,^5^,^6^,^7^ allows unbiased sampling and unambiguous three-dimensional (3D) localization of up to thousands of neurons. Because of the recent introduction of acousto-optic (AO) random-access scanning^8^,^9^,^10^,^11^, it has also become technically possible to rapidly scan such large populations in two and three dimensions.

However, the maximal number of neurons from which workable functional signals can so far be obtained (a few hundred at best) is at least an order of magnitude smaller than what the current state of the technology allows to scan, because the signal-to-noise ratio (SNR) of the recorded fluorescence drops with the number of recorded cells. Indeed, action potentials (spikes) need to be extracted from the recorded fluorescence changes of a synthetic or genetically encoded (GECI) calcium (Ca^2+^) indicator^12^,^13^. Single spikes lead to intracellular Ca^2+^ increases with fast rise- but slow decay time (time-to-peak ∼8–40 ms, slightly longer in case of certain GECIs; decay constant ∼0.3–1.5 s (refs 10, 13, 14)), causing the transients induced by individual spikes to overlap, often adding up nonlinearly^15^. Moreover, the signals are often contaminated by large noises, including by baseline fluctuations similar to the actual responses. Therefore, accurately reconstructing spikes from noisy calcium signals is a critical challenge on the road to optically monitoring the firing activity of large neuronal populations.

Numerous methods have been proposed for estimating the spiking activity^10^,^16^,^17^,^18^,^19^,^20^,^21^,^22^,^23^,^24^,^25^,^26^,^27^. However, none tackles all three of the following critical challenges: first, finding the optimal spike train is algorithmically challenging. In popular spike estimation methods based on template matching^10^,^16^,^17^,^18^,^19^,^20^,^21^,^22^ the time needed to find the optimal spike train underlying a recorded fluorescence time series grows exponentially with its number of time points, just like the number of possible spike trains. To make computation costs affordable, approximations become necessary, thus curbing estimation accuracy. Second, the baseline fluorescence level often fluctuates. Third, model parameters (for example, the unitary Ca^2+^ fluorescence transient's amplitude *A* and decay time *τ*) are inhomogeneous across neurons and cortical areas. As a consequence, often only spiking rates or -probabilities are extracted from Ca^2+^ signals, rather than the individual spikes^24^,^25^,^26^,^27^,^28^,^29^,^30^,^31^. Despite the advantages of determining such ‘activity levels' at low SNRs, lacking the actual spike trains hampers investigating temporal coding, causal network relations and the like.

Our method ‘MLspike' tackles the first two challenges by finding the most likely spike train underlying the recorded fluorescence using a maximum-likelihood approach. An ‘auto-calibration' procedure addresses the third one.

We tested MLspike (algorithm and autocalibration procedure) on extensive simulations and on real biological data consisting of 55 neurons from seven different preparations, and we gauged it against four state-of-the-art algorithms. The first one, Peeling^10^, provides a unique spike train as does MLspike. The other three algorithms provide spiking probabilities or rates, namely the Sequential Monte-Carlo (*SMC*) method published in^24^ and the recently published Constrained Deconvolution (CD) and Markov Chain Monte-Carlo (MCMC) algorithms^26^,^27^. All these algorithms were compared with MLspike on our biological data set, while we chose only Peeling for benchmarking on the synthetic data because of the type of its output (that is, spikes rather than spiking rates) that makes the comparison more straightforward, its recently published extensive simulations and quantifications against noise^32^ and its robustness against baseline drifts.

## Results

### Algorithm

**Figure 1:**
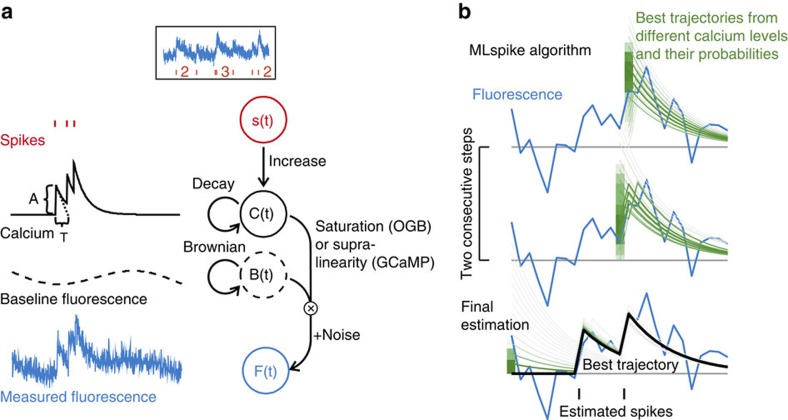
Model and algorithm. (**a**) Physiological model. Upon emission of *s(t)* spikes, intracellular Ca^2+^ concentration *C*(*t*) is driven by an increase *A* (the unitary calcium response) × *s(t)*, then decays to the resting value with time constant *τ*. The measured fluorescence *F(t)* is the product of a drifting baseline fluorescence *B*(*t*) with a nonlinear function of *C*(*t*) accounting for dye saturation and GCaMP nonlinearities; a noise term is added. Note the similarity between the resulting trace (blue) and real fluorescence data (inset; numbers adjacent to spikes indicate their multiplicity). (**b**) ‘MLspike' algorithm illustrated on a schematic example without baseline drift. (top and middle) The probabilities (white-green colour code) of ‘best trajectories' originating from all possible calcium values (*y* axis, for display purposes same scale as fluorescence) at time *t* (*x* axis) are calculated, iteratively for decreasing time. (bottom) Once time zero is reached, the best Ca^2+^ trajectory uniquely defines the ‘maximum posterior' estimated spike train (bottom) (see Methods and Supplementary Movie).

MLspike's key features consist in using a physiological model (Fig. 1a) of both intracellular Ca^2+^ dynamics and baseline fluorescence—which turned out to be a key step for accurate estimations on real data—together with a filtering technique that runs in linear time. The framework is general and the model is thus easily modifiable to incorporate additional physiological details. In contrast to previous hidden Markov model approaches^24^,^26^,^27^ that yield spiking probabilities, -rates or distributions of spike trains, MLspike provides the unique spike train that maximizes the likelihood of obtaining the recorded fluorescence time series. To do so, we use a version of the Viterbi algorithm^33^ to estimate the optimal input (the spike train) by maximizing an *a posteriori* (MAP) distribution probability (Fig. 1b and Supplementary Movie); for MAP estimation from calcium signals in another context see (ref. 34).

Briefly, the concept underlying MLspike is to calculate, iteratively for decreasing times *t*: the set of most likely Ca^2+^ trajectories starting from all possible Ca^2+^ values (*y* axis) at time *t*, and the relative probabilities of these trajectories (Fig. 1b, green colour code). A conditional probability maximization then allows to step from time *t* (top) to *t*−1 (middle), and once time zero is reached, the most likely trajectory defines a unique ‘maximum posterior' spike train (bottom). Importantly, for a given *t*, the set of most likely Ca^2+^ trajectories has to be calculated only once, thus ‘collapsing' together trajectories that pass through the same point(s). As a result, the number of trajectories to evaluate grows only linearly with the number of time points, rather than exponentially.

The classical Viterbi algorithm applies to a discrete state space. We were able to generalize it to a continuous one by discretizing the state space and by interpolating at each time step (see Methods for details). This allowed us to gain in speed and in accuracy for the representation of probability distributions as compared with particle filter representations^24^ or to Metropolis–Hastings sampling^26^.

### Benchmark on simulated data

We first benchmarked MLspike on simulated data, assuming known model parameter values. We quantified error rate (ER) as 1—F1-score (that is, 1—harmonic mean of sensitivity and precision), which amounts to an average of the percentages of misses and false detections biased towards the worst of the two, see ref. 32 and Methods. Noise level was defined as the noise root-mean-square (RMS) power in the 0.1–3 Hz frequency band, normalized by *A*. As we shall see, this quantification reflects the fact that low- and high-frequency noise weakly affects estimation accuracy.

**Figure 2:**
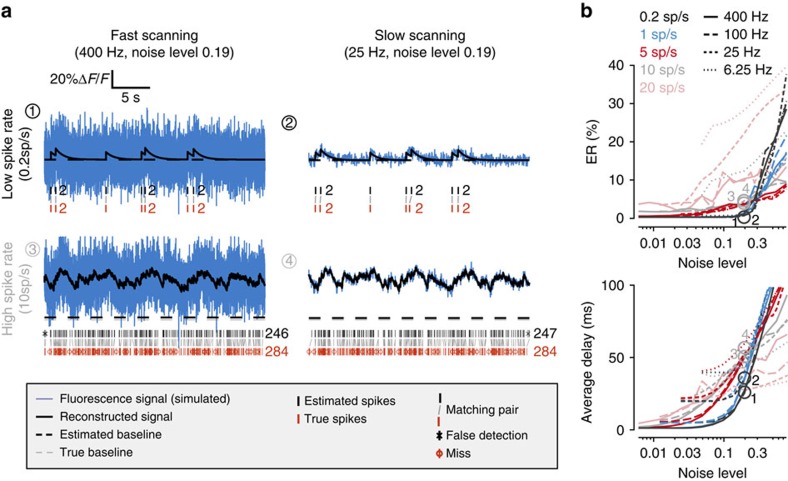
Simulations with flat baseline. (**a**) Four example spike reconstructions at high and low spiking rates and frame rates. Note how an accurate baseline level estimation (unknown, well below the signal) warrants good performances even at high spiking rates. (**b**) Error in spike estimation (ER) and timing (defined here and elsewhere as the average of the absolute value of the delay between an estimated spike and the corresponding true one), as a function of noise level, defined as RMS/A restricted to the 0.1–3 Hz frequency range. Here and elsewhere, numbered circles on curves mark the position of correspondent example traces. Different colours and line styles denote different spiking- and frame rates. The legend below **a** defines the meaning of the remaining symbols used here and throughout the article. For further characterization of the estimation error (misses, false detections, timing, dependence on parameter values) see Supplementary Fig. 1. For a benchmark against Peeling, see Supplementary Fig. 2.

We began by characterizing ER as a function of white (that is, photonic) noise (Fig. 2). Although the baseline was flat, its ‘level' was unknown to the algorithm, which had to recover it. ER remained below 1% up to noise levels of 0.2 (top left), except for high spiking rates (bottom left), as is expected given the long Ca^2+^ transient decay. Frame rate impacted little on ER, implying that what matters for spike detection is the total amount of fluorescence captured per unit time, rather than sampling rate. In contrast, high frame rates obviously improved timing accuracy, especially at low noise levels (Supplementary Fig. 1). We also benchmarked MLspike against Peeling, yet in a slightly simpler situation, that is, with provided baseline level, thus reproducing the published results^32^ using the code available online (Supplementary Fig. 2). With those settings, MLspike outperformed Peeling by ∼20%.

**Figure 3:**
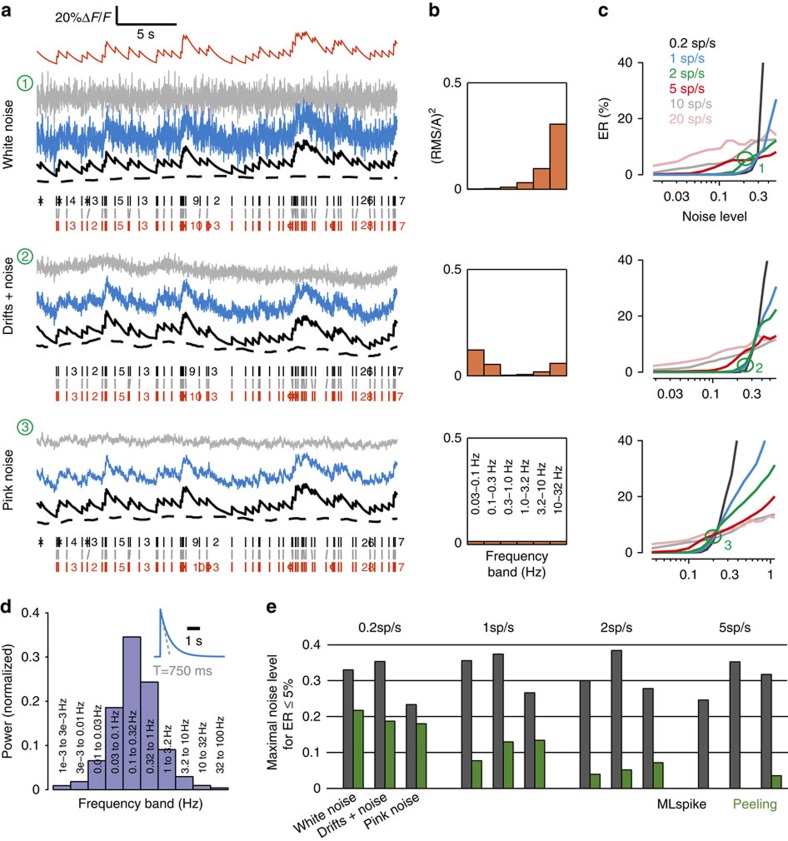
Simulations with constant, drifting and fluctuating baseline. (**a**) Examples of estimations with similar noise level but different noise types. Blue traces: sum of red (noise-free fluorescence signals) and grey (noise) traces. Mean spike rate: 2 sp s^−1^. (**b**) Power spectra of noise in **a**. (**c**) ER corresponding to the noise types in **a**,**b**, as a function of noise level and spiking rate. To facilitate comparison, abscissae were shifted such as to vertically align the three graphs on identical SNR values: note that, at equal SNR, pink noise has a higher noise level than white noise and thus a larger ER. (**d**) Power spectrum of the function used to model the fluorescence response evoked by a single spike (inset). Most of it falls into the frequencies between 0.1 and 3 Hz, which explains why noise in this frequency band has such a prominent effect on the algorithm's performance and justifies our definition of the noise level. (**e**) Overall performance comparison between MLspike and Peeling, for the three noise types. Bars represent maximal noise levels at which spikes are estimated with ER≤5% (top) or ≤10% (bottom), at different spiking rates. The difference between the two algorithms was particularly large at higher spiking rates (comparisons at even higher rates were not possible due to failures of Peeling). For further characterization of the estimation error, see Supplementary Fig. 3. For a benchmark against Peeling, see Supplementary Fig. 4. Frame rate: 100 Hz in all panels.

Next, we characterized MLspike*'s* performance with respect to the noise's frequency spectrum (Fig. 3a–c and Supplementary Fig. 3): white noise, low-frequency drifts (that is, slow baseline fluctuations) together with white noise, and pink noise (which has equal power in all octaves and includes complex baseline fluctuations and photonic noise). As expected, pink noise induced by far the largest ERs when noise was quantified by RMS power calculated over the entire frequency spectrum (Supplementary Fig. 3). However, when noise was quantified by RMS power restricted to the 0.1–3 Hz frequency range, MLspike handled all noise types similarly (Fig. 3c). This reflects the fact that the critical noise frequencies are those that fall within the dominant part of the calcium fluorescence response spectrum (Fig. 3d) and justifies our quantification of noise level. MLspike was then benchmarked against Peeling in extensive additional simulations, largely outperforming it throughout all noise types and levels (Fig. 3e, for details including parameter value explorations see Supplementary Fig. 4). Importantly, in the case of spiking rates of 5 sp s^−1^ and higher, MLspike could accurately estimate (for example, at the ER ≤5% threshold) spike trains in the presence of ∼10 times more noise than Peeling. This underscores one of MLspike*'s* main advances with respect to current state of the art: its capability to handle not only high noise levels but also dense firing patterns (up to 20 Hz), where fluorescence rarely decays back to baseline.

**Figure 4:**
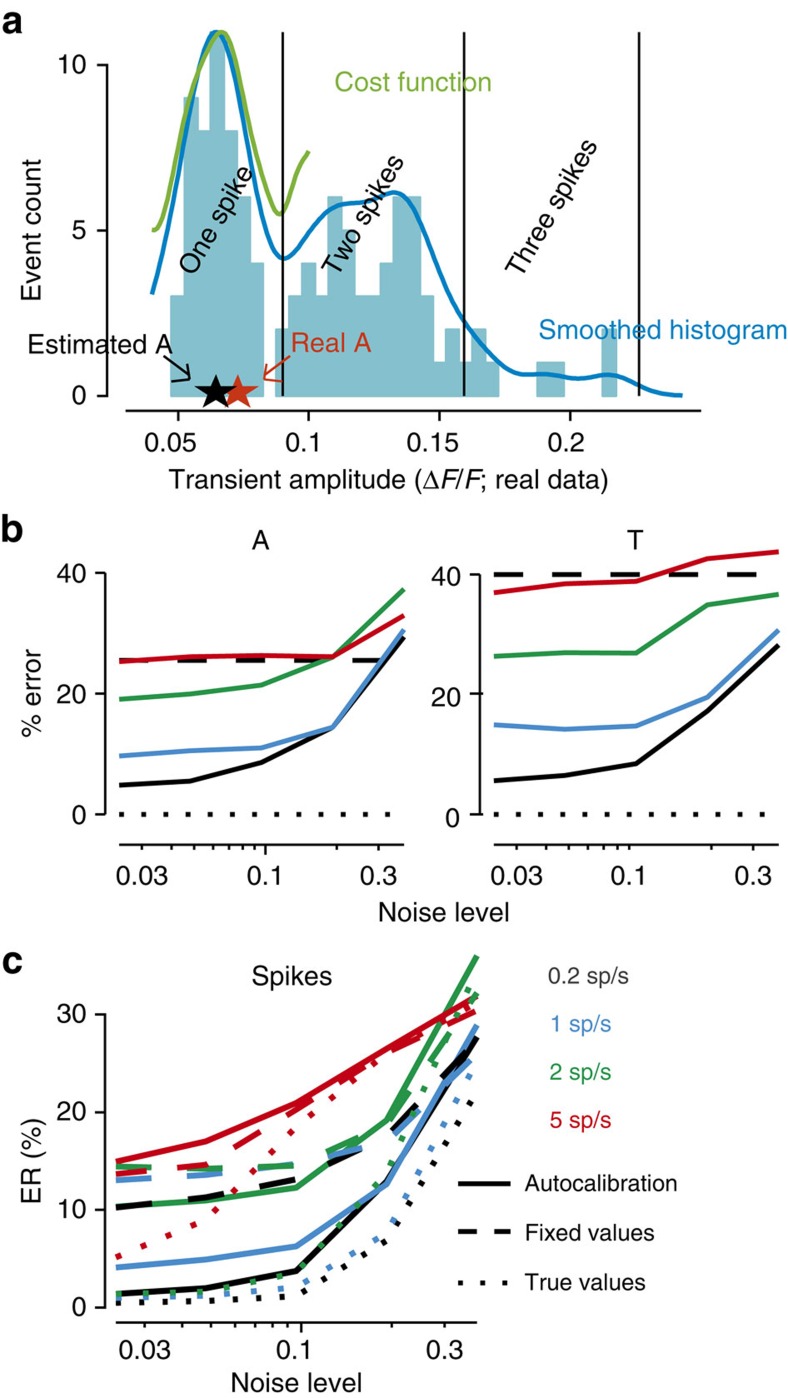
Autocalibration algorithm. (**a**) Example estimation of parameter *A* via autocalibration on signals recorded *in vivo* from a neuron in rat somatosensory cortex (bulk-loaded with OGB). The estimation is based on a histogram of the detected isolated fluorescence transient amplitudes, yielding a specific cost function that allows the assignment of a number of spikes to each transient amplitude. For details, see text, Methods and Supplementary Fig. 5. (**b**) Mean error in estimating *A* and *τ* upon autocalibrating (solid) the model parameters on a population of simulated fluorescence signals with *A* and *τ* drawn from a log-uniform distribution (4%<*A*<10% and 0.4s*<τ*<1.6 s), as compared with using their true values (dotted) or fixing the parameters to their median (dashed; note the larger error on *τ* as compared with *A* in this last case, resulting from its larger initial variability range). (**c**) Same as **b** but for mean ER of estimated spike trains. Frame rate: 100 Hz.

All above simulations were generated using the same model parameters values (*A*=10%, *τ*=1 s), as is commonly done by using the same ‘good estimate' parameters for all cells as. However, simultaneous electrophysiological and fluorescence recordings both of ourselves and others^10^,^20^, show remarkable variability among cells recorded using the synthetic calcium indicator Oregon Green BAPTA-1-AM (OGB): *σ*_A_/<*A>≈*30-40% and *σ*_*τ*_/<*τ>≈*40-50%. In the case of GECIs, the variability can be even larger. Neglecting it obviously reduces estimation accuracy. Therefore, we developed an original ‘autocalibration' method that estimates *A*, *τ* and *σ* (a parameter accounting for noise level) for each neuron, directly from its recording. In contrast to previous work^20^,^24^,^26^,^27^, our method takes advantage of *a priori* knowledge of each parameter's specific characteristics. In particular, the estimation of *A* relies on the discrete nature of spikes and thus of the amplitudes of isolated Ca^2+^ transients (Fig. 4 and Supplementary Fig. 5); *τ* is easily estimated by single-exponential fitting because it governs the shape of the transients and *σ* is heuristically determined as a function of the signals' spectral content (see Methods). The remaining model parameters, namely saturation, baseline drift and spiking rate were found to impact less on the estimation and were thus assigned to fixed values. In the GECI's case, the saturation parameter was replaced by two parameters coding for supra-linearity.

Autocalibration was tested at multiple noise levels on simulated fluorescence signals with *A* and *τ* drawn from a distribution reflecting the statistics of our data acquired using OGB (*τ*=0.81±0.40 s, *A*=5.2±1.6%, *n*_cells_=24). Even when run on as few as three 30 s long trials, autocalibration yielded satisfying estimates for *A*, *τ* and *σ*, at noise level up to 0.2 (Fig. 4b). Importantly, the estimates obtained using ‘autocalibrated' parameters were much more accurate than using ‘good estimates', closely approaching the level obtained using the true simulation parameter values (Fig. 4c).

Obviously, autocalibration performance decreased with increasing noise and spike density, mostly because the heuristics used to estimate parameter *A* becomes less appropriate (Fig. 4b). Indeed, for noise levels above ∼0.2 or spiking rates above ∼5 spikes per second autocalibration did not perform better than using fixed parameter values (Fig. 4c). For practical usage, in the Supplementary Note 1 (‘Factor Box') we provide an intuitive, example-based analysis of how both MLspike's and autocalibration's estimation accuracies depend on a multitude of factors, including primary (for example, frame- and spiking rate) and secondary ones (for example, calcium indicator choice), and their interaction with our method's internal parameters.

### Performance on real data

**Figure 5:**
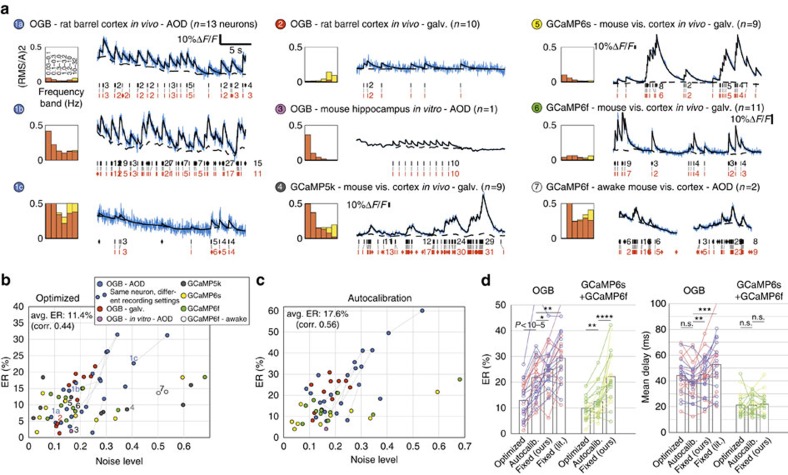
Application to seven different sets of real data. (**a**) Representative examples of nine neurons characterized by different mean spike rates and noise types. Examples were drawn from data gathered using different scanning methods, Ca^2+^ indicators, cortical area and preparation (*in vitro, in vivo*, anaesthetized and awake). The slow decay in examples 1a–c indicates that the AODs transmission efficiency had not yet settled (recording still within the ‘warm-up' period). Note the different vertical scale for different Ca^2+^ indicators. (left) Noise power spectra, separated into photonic- (yellow) and non-photonic components (orange). (**b**,**c**) Performance of MLspike on 66 recording sessions (55 neurons, frame rate: 30–200 Hz), plotted as a function of noise level, using (**b**) ‘optimized' parameter values obtained from the simultaneous electrical recordings (such as to best predict measured calcium time courses from the recorded spikes) or (**c**) autocalibrated values. Note the larger correlation between ER and noise level in the latter case, due to its effect on both parameter- and spike-train estimation. (**d**) Performance comparison (left, ER; right, mean temporal error) of MLspike run on both OGB- and GECI data (*n*=24 and 20, respectively), upon using different parameter choices: optimized, autocalibrated and a fix parameter obtained either by averaging the optimized values from our data, or by using literature value. In the GECI case, we could not use the literature value^13^ because of a different normalization convention than ours (division of the background-subtracted signal by baseline—0.7 × background, rather than by the baseline alone), resulting in slightly larger values for parameter *A*. Points indicate average ER for each individual neuron (same colour code as in the other panels). Stars indicate statistical significance (one-sided Wilcoxon signed ranked test, *: *P*<0.05, **: *P*<0.01, ***: *P*<0.001, ****: *P*<1e−4). For more details on parameter estimations, noise level quantification and GCaMP model, see Supplementary Figs 6,7 and 9.

**Figure 6:**
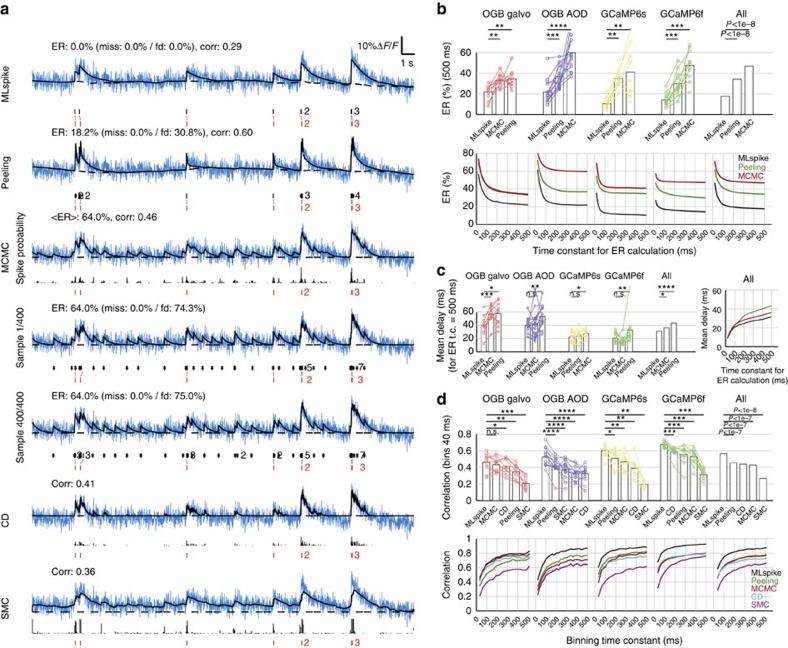
Benchmarking against state of the art on real data. (**a**) Example estimations on the same recording (OGB) using MLspike, Peeling, MCMC, CD and SMS algorithms. MLspike and Peeling estimate a unique spike train, therefore ER value can be computed based on misses and false detections, while the other algorithms estimate spiking probability (up to a scaling factor in the case of CD). In the case of MCMC, this spiking probability is obtained from 400 sample spike trains (the first and last are displayed as well), from which an average ER value can be computed. Correlations between true spike train and estimations are also displayed. Each algorithm was run using the parameter values obtained with its own autocalibration procedure, except Peeling, which was run using a fix set of parameters (OGB: literature, GECIs: mean optimized from our data). See Supplementary Fig. 8 for two additional examples. (**b**-**d**) Comparisons of the five algorithms' performance on the whole population, separately for each data set and on all data pooled together (same graphic conventions as in Fig. 5d). (**b**) First line shows performance quantification as mean ER using a spike-assignment time constant of 500 ms (an estimated spike was considered as correct if there was a yet unassigned recorded spike <500 ms away). Second line displays the mean ER as a function of correspondence window. (**c**) Spike estimation delay (mean temporal error) obtained using the different algorithms. The rightmost graph plots the delay as a function of spike assignment time constant. Note that even for time constants down to ∼50 ms the mean temporal error was much lower than the maximally allowed one. This difference obviously decreased for very small time constants and finally converged to the maximal allowed value of 10 ms for a 20 ms time window. (**d**) Same comparisons as **b**, but using correlation as a measure of estimation accuracy rather than ER.

Next, we tested the performance of our method on real data acquired in multiple brain areas (barrel cortex, V1 and hippocampus), species (rat and mice) and preparations (*in vitro* and *in vivo*, anaesthetized and awake), obtained using either the synthetic Ca^2+^ dye OGB or last-generation GECIs (GCaMP5k (ref. 15) and GCaMP6s/GCaMP6f (ref. 13)) (Figs 5 and 6), Supplementary Figs 6 and 7). The GCaMP data were either obtained by the authors themselves (awake mouse), or taken from a public repository (anaesthetized mouse, see Acknowledgements). Actually occurred spikes were recorded electrically in cell-attached mode, simultaneously with the Ca^2+^ fluorescence. We first assessed MLspike's accuracy independently from performance drops due to wrongly estimated model parameters: for each cell, physiological parameters *A* and *τ* were first ‘calibrated', that is, adjusted so as to best predict measured calcium time courses from the recorded spikes (Supplementary Fig. 6), then noise and drift parameters QUOTE and *η* were ‘optimized' by minimizing ER with respect to the simultaneous electrical recordings (Fig. 5a,b and Supplementary Fig. 7a). This yielded the estimations we refer to as ‘optimized' throughout this work. Average ER was of 11.4% (OGB: <ER>=12.8% and ER<20% in 83.3% of the cases; GCaMP6s+GCaMP6f: <ER>=9.8% and ER<20% for all cells), fast spiking (Fig. 5a, second example: mean firing rate 5.4 sp s^−1^) and noisy neurons yielding the higher values (correlation *ρ*=0.44 between noise level and ER, Fig. 5b). Noise level (which is normalized by *A*), was inhomogeneous due to variability in cell-specific amplitude of fluorescence transients, staining, Ca^2+^ indicator, preparation, physiological condition and scanning technology—galvanometric or AO deflectors (AOD) based (Supplementary Fig. 6a,b). Laser intensity and the number of simultaneously imaged neurons (between 20 and 1,011) more specifically affected the photonic part of the noise (yellow in the spectra).

We then ran the estimations again, this time using parameter values autocalibrated from the fluorescence data themselves, rather than calibrated using the electrical recordings (Fig. 5c and Supplementary Fig. 7b,c). Strong correlations were obtained between autocalibrated and optimal *A* and *τ* values (Supplementary Fig. 7c), in particular for OGB and GCaMP6s (*ρ*=0.5 and 0.9 for *A* estimation, *ρ*=0.8 and 0.57 for *τ* estimation), while autocalibration was more difficult on GCaMP6f signals (*ρ*=0.53 for *A*, 0.13 for *τ*), possibly because of the small amplitude of individual spike responses. Average ER on spike estimations equalled 17.6% (OGB: <ER>=21.8%, ER<20% in 45.8% of the cases; GCaMP6s+GCaMP6f: <ER>=12.5% and ER<20% in 85% of the cases). These estimations proved more accurate than when using fixed ‘good estimate' parameter values (Fig. 5d, left). In that case, using our average calibrated values for *A* and *τ* yielded an <ER> of 25.1% for OGB (<20% in 20.8% of the cases; when using values from the literature^10^ instead: <ER>=29.2%, ER <20% in 25% of the cases) and of 22.2% for GCaMP6 (<20% in 45% of the cases). In terms of temporal precision, in the case of OGB, MLspike combined with autocalibration performed best (Fig. 5d, right). In the case of GCaMP6, all estimations yielded comparable results (the better temporal precision obtained on the GCaMP6 data set as compared with the OGB one is probably the consequence of a lower average noise level, rather than an indication of specific differences between indicators). The optimal noise-level bandwidth could be satisfactorily approximated to 0.1–3 Hz for both OGB and GCaMP6, despite some small differences between the two (Supplementary Figs 7d,e).

Using the same data as above, we extensively compared the performance of MLspike (with autocalibration) to that of four other state-of-the-art algorithms, namely Peeling, MCMC, CD and *SMC* (Fig. 6 and Supplementary Fig. 8). To do so also for methods yielding spiking rates rather than individual spikes (MCMC, CD and *SMC*), we quantified the estimation error of all algorithms using the correlation between the measured and the reconstructed firing rate time series as in ref. 27 (bin=40 ms). In addition, we also compared the algorithms yielding actual spikes (MLspike, Peeling and representative realizations of spike trains estimated by MCMC), by quantifying the estimation error using ER, which, as opposed to the correlation metric, is not invariant for affine transformations of the unitary fluorescence response *A*. The example in Fig. 6a conveniently illustrates this shortcoming of the correlation measure: MLspike finds the most accurate spike train (compare ER, <ER> and actual spikes), yet its estimation accuracy is ranked inferior to that of the other algorithms when quantified using correlation.

In Fig. 6b–d, we compare the performance of the five algorithms on our various data sets. MLspike clearly outperformed the other algorithms, both when using ER or correlation as a measure for accuracy (Fig. 6b,d, respectively). We also tested temporally more restrictive criteria for assigning estimated spikes to measured ones in the calculation of ER (from the default coincidence window of 500 ms down to 20 ms) and using different bin sizes when calculating the correlation (20–500 ms). As expected, decreasing these time constants reduced estimation accuracy for all algorithms; yet, MLspike remained the most accurate one at all tested temporal tolerances (Fig. 6b,d, bottom). Finally, we also compared the estimated spikes' timing accuracy, first with fixed ER time constant (500 ms) and then by varying it and calculating the average resulting temporal error (Fig. 6c left and right, respectively). Also here, MLspike was more accurate than all the other algorithms, at least on the grand average. Importantly, the mean temporal error was always several times smaller than the maximally accepted temporal tolerance; for instance, the spikes estimated with a tolerance window of 50 ms had, in the average, a temporal error of only ∼15 ms (Fig. 6c, right).

The specific choices made for the physiological models underlying MLspike*'s* estimations appear to be largely responsible for its superiority over other algorithms—at least on this data set. For example, Fig. 6a and Supplementary Fig. 8a show how the MCMC, CD and *SMC* algorithms that do not explicitly model baseline drifts tend to explain those with (incorrectly placed) spikes. In the case of Peeling (which does estimate baseline drifts), the worse performance is rather due to a less sophisticated statistical approach. Finally, the inclusion of nonlinearity in the model turned out to be crucial to correctly handle the responses in case of GECIs (Supplementary Fig. 8b).

To account for the supra-linearity of GECIs, we used a heuristic, cubic polynomial based, response model^15^ (Supplementary Fig. 6). Indeed, somewhat surprisingly, performance did not improve significantly (although temporal accuracy did) when two more physiological models were used instead, one of which included finite, computationally more expensive, rise times (see Methods, Supplementary Fig. 9). This underscores the importance of further efforts aimed to account more accurately for the dynamics of GECIs.

### Benchmark on data recorded simultaneously from 1,000 neurons

**Figure 7:**
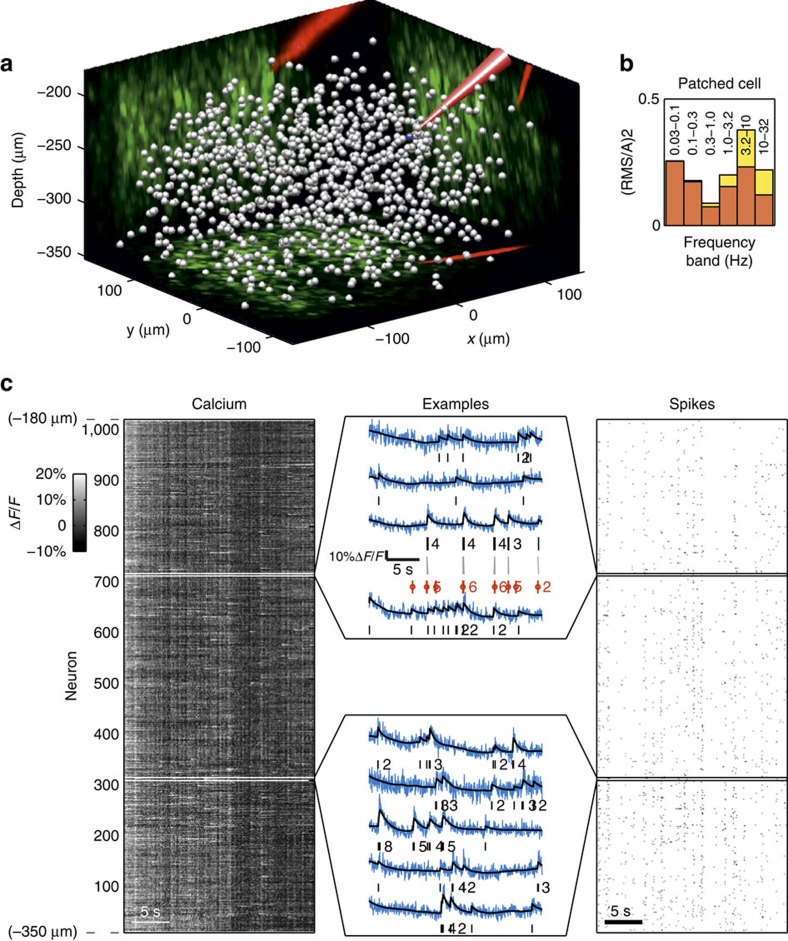
Application of MLspike to 1,000 recorded neurons. (**a**) Volumetric reconstruction of 1,011 recorded neurons (grey), with *x*,*y*,*z* projections of z-stack (green) and the cell-attached patch electrode (red) for simultaneous electrical recordings from an individual cell (blue). (Recording effectuated with AOD scanning in anaesthetized rat barrel cortex stained with OGB-1-AM.) (**b**) Noise spectrum for the patched neuron, separated into photonic- (yellow) and non-photonic components (orange). (**c**, left) Grey scale display of the imaged neurons' *F/F* responses. Zooming into the responses (middle, one zoom includes the patched cell) clearly shows cell-specific spiking patterns. (right) Raster display of the spikes from the 1,011 neurons obtained with MLspike in combination with the autocalibration procedure. Although the recorded fluorescence, the spike reconstructions and their confirmation by the simultaneous electric recording show correlated activities in the network (appearing as vertical alignments of spikes), the detailed inspection of individual neurons' firing (middle) clearly reveals independent, cell-specific, activity patterns.

Since the combination of the autocalibration method and the MLspike algorithm allows more accurate spike estimation than so far, lower SNR levels in the raw data become acceptable. This allows to take better advantage of current AOD-based two-photon random-access laser scanning technology for very large population imaging in 3D (ref. 11). As a proof of concept, we recorded signals from 1,011 cells randomly distributed within 300 × 300 × 170 μm^3^ (Fig. 7a), at 30 Hz frame rate. Again, a cell was patch-recorded simultaneously with imaging. Once more, its noise power spectrum (Fig. 7b) shows that the photonic contribution to noise in the critical bandwith (0.1–3 Hz) is small.

Figure 7c shows the raw signals and raster plots of the spikes estimated from the 1,011 neurons. The recurrent vertical stripes visible at the global level (left and right panels) mark the presence of correlated network activity, consistently with the presence of slow collective oscillations (up-and-down states) that are known to occur under various circumstances in the anaesthetized preparation^35^, and in particular in the rat under Urethane anaesthesia^35^,^36^. At a more detailed level, closer inspection (Fig. 7c, middle) shows clear differences between the fluorescence traces recorded from different neurons, and the same is true for the estimated spike trains.

The patched neuron allowed assessing estimation accuracy, yielding an ER of 26%, which is clearly better than the ER obtained when we fixed parameters to their mean values (36%), or when we used MCMC (42%) or Peeling (57%) (CD and SMC performing even worse, although using a correlation-based measure). Even at the current proof-of-concept level, such an accuracy improvement is highly relevant for the determination of network connectivity (for example, following the theoretical study of ref. 32, it would result in a gain of 1.2–2 in hub cell hit-rate with respect to current state of the art).

## Discussion

MLspike achieves model-based spike inference to reconstruct the MAP spike train that best accounts for measured fluorescence signal. With respect to current state of the art, MLspike divides the estimation error by an average factor of ∼2 (Fig. 6), and much more in specific contexts such as high (⩾2 sp s^−1^) spiking rates (Fig. 3e). Other advantages compared with more *ad hoc* methods such as the Peeling algorithm are a well-posed mathematical definition of the problem, a small number of parameters (in particular thanks to our model reparameterization, see Methods), and flexibility with respect to changes in the underlying model.

A simple, yet critical, novelty of our model formulation compared with all previous methods is the inclusion of neuron-specific fluctuations in the baseline fluorescence. Such baseline drifts and -fluctuations are often encountered *in vivo*, but even *in vitro*; they also appear during the first ∼15 s of AOD operation or even later, thus requiring the introduction of a ‘warm-up' period before each data acquisition; they might also reflect slow calcium concentration changes not related to the cell's spiking activity. It is particularly in the context of drift- and fluctuations-containing signals that MLspike outperformed Peeling (simulations in Supplementary Fig. 2 versus 4), as well as in the case of so-far untreatable spiking rates up to 20  sp s^−1^ (Figs 2 and 3).

With respect to the modelling of GCaMP6 nonlinearities, somewhat surprisingly, polynomial and physiological models appeared to perform similarly. While this calls for further modelling of the underlying processes^37^, it also underscores the adequacy of simple phenomenological models with few parameters, as long as they capture the main features of the underlying physiology.

Recently, estimations based on machine learning techniques have been proposed^31^. Since they extract their inference parameters directly from the learning data set, such model-free methods could in principle learn by themselves how to ignore drifts or other confounding effects. However, they require an adequate choice of learning data set. Moreover, at present they yield only spiking rates, rather than actual spikes; even less have they be proven to be able to autocalibrate, that is, to adapt their internal kernels to the individual statistics of each neuron. Conversely, the advantage of model-based approaches is their robustness in establishing a set of possible dynamics, parameterized by well-defined quantities (*A*, *τ*, ...), which allows to adapt the estimation to each neuron's characteristics, rather than using average parameter values.

A number of methods estimate spiking probabilities or instantaneous spiking rates^24^,^25^,^26^,^27^,^28^,^29^,^30^,^31^. This approach has advantages when used on data that clearly lack single-spike resolution or when it is important to assess the uncertainty of the estimation. However, when it comes to investigating temporal coding and causal network relations, estimating the optimal time series of the spikes themselves, as do MLspike, Peeling and others, can be advantageous. From the practical point of view, it should also be noted that dealing with a single spike train has the advantage of being able to use—essentially *as-is*—the large thesaurus of standard methods available today for spike train analysis.

Importantly, when investigating network properties, the tolerable jitter on the estimated spikes' timing is considerable (beyond 25 ms in a recent study on synaptic connectivity^38^), thus relaxing the constraints on temporal accuracy with respect to electrophysiological standards. Furthermore, the ongoing progress in both fluorescent marker- and imaging technology is likely to make robust and precise single-spike estimation increasingly accessible^13^.

Conversely, both approaches can be used to investigating rate coding or average responses. Importantly, in our hands, MLspike (and in most cases also Peeling) outperformed MCMC, CD and the *SMC* method also at estimating instantaneous firing rates/probabilities (in the former case calculated from the estimated spikes, in the latter case deduced from the distribution of estimated spike trains or directly extracted from the calcium fluorescence). Part of this difference is likely due to MLspike*'s* (and Peeling*'s*) better handling of baseline drifts, underscoring the importance of this feature.

Interestingly, the MCMC algorithm^26^,^27^ returns actual sample spike trains that can be used, for example, to investigate network properties based on spike times. At the same time, MCMC returns many such spike trains, sampled according to the posterior probability distribution, thus allowing both the estimation of spiking probabilities and an indication of the level of estimation uncertainty. Similarly, we have adapted MLspike so it can return, upon user choice, the spiking probability distribution, or a set of spike trains sampled according to the posterior distribution, rather than the unique MAP spike train (see Methods and Supplementary Fig. 10). Caveats apply, however, to the interpretation of such sample spike trains. For instance, the ‘variability' observed in sample spike trains can be erroneous (and therefore misleading), either because the algorithm is not robust enough to avoid local maxima, or, more importantly, because of systematic errors. Those can result, for example, from a mismatch between the used response model and the data. Such a situation can be seen in Fig. 6a (and Supplementary Fig. 8): different sample spike trains returned by MCMC tend to reproduce the same estimation errors but are very similar one to another. The resulting low variability would make the user overly confident with respect to the quality of the reconstruction.

In terms of algorithm and implementation, MLspike implements a Viterbi algorithm to estimate a MAP spike train from calcium signals, for the first time to our knowledge. Additional novelties include the representation of probability distributions as discretized onto a grid, as in histogram filters, so they can easily be spline-interpolated over the whole state space (as opposed to, for example, particle representations as in ref. 24). This is critical for computing the MAP estimate, and also contributes to faster computations (at least up to state-space dimensions <4). For additional details, including simplifications that further increase computation speed, see Methods.

The need to further improve the response model may increase the number of its dimensions and with it the dimensionality of the grid onto which probabilities are represented. This would result in a prohibitively large number of points at which the probability has to be calculated. Luckily, however, this probability would be non-negligible only within a thin subspace of the grid, which opens the door to further improvements of our method by using sparse representations, in order to computationally streamline also versions with a higher dimensional (>3) response model. A possible implementation would be to compute, iteratively, the probabilities for time (*t*−1) only for the states (that is, grid points) that can be parents of states represented at time *t*, and to set a probability threshold that determines which states to represent and which not at each time-step.

Our autocalibration procedure is somewhat more *ad hoc*. Yet, it runs fast and shows that estimating each cell's model parameters from its own raw signals—even at current error levels—yields more accurate spike train estimates than using fixed parameter values (population average or from the literature). Other, more well-defined, methods maximize the likelihood of the fluorescence signals^24^,^25^,^26^,^27^,^28^,^29^, but such optimization is computationally more expensive. Moreover, these methods do not include any *a priori* on the cells' parameters. This is not the case of our autocalibration, which uses such information by allowing only a range of values for certain parameters (for example, *A*), and even clamps some others to fixed values (such as those governing nonlinearities, which are particularly difficult to estimate). Such *a priori* can prove advantageous in situations with noisy or little data, or when only few isolated spikes are available. Ideally, autocalibration methods would be able to combine such information with that provided by the data itself.

The open source code of our method is available as Supplementary Software and includes introductory demos. A number of practical considerations aimed at understanding the principles and limitations of spikes estimation, such as the concept of ‘noise level' we introduced, can be found in the ‘Factor Box' (in Supplementary Note 1). It qualitatively but simply and intuitively illustrates how to adjust the few parameters of MLspike in the rare cases that the default values should be inadequate (for a quantitative and systematic study of parameter dependencies, see Supplementary Figs 1–4).

Our novel method makes it possible to optimally exploit the capabilities of current hardware. Warranting more accurate spike train extraction from larger sets of cells than so-far is a step forward in the investigation of local network properties, such as temporal coding (at very high SNR) and correlations (at the single-spike level or, at lower SNR, at the level of changes in spiking rate). It also extends the applicability of two-photon imaging to investigating more densely connected networks than so-far, improvements in the determination of functional connectivity (for a quantitative analysis see ref. 32: the hub-cell hit rate) and network topology (for example, of power-law versus log-normal type^39^,^40^,^41^). Importantly, the constraints on timing accuracy are relatively affordable in this context (for example, using iterative Bayesian inference it has been shown that network synaptic connectivity and flow direction can be predicted even if spikes are encoded at a precision of 25 ms and below^38^).

These perspectives are especially interesting when the Ca^2+^ probes are expressed in genetically modified strains^14^,^42^, where the imaged volume is not limited by the spatial spread of extrinsic fluorescent markers. Recent progress in waveform shaping^43^,^44^ that corrects for scattering-induced deformations should also allow a significant extension of the volume accessible for imaging, into depth in particular.

Recently, more general approaches have been proposed, aimed to jointly infer regions of interest and spikes^23^,^27^,^45^,^46^. Although the strength of these methods resides in exploiting the full spatio-temporal structure of the problem of spike inference in calcium imaging and in offering an unbiased approach for ROI determination, they have the disadvantage of requiring that the full two-dimensional (2D) or 3D data are available, which is not the case in random-access scanning. Indeed, there, one scans only the points of interest—albeit at 3D and at much higher speeds, for instance using AOD-technology^11^. Nevertheless, MLspike could straightforwardly be added to the list of available spike estimation algorithms even in algorithms of these kind^27^, thus increasing their data processing power.

Finally, we have shown that it is straightforward to modify our method to include different response models—here, to account for the specific nonlinearities of GECIs. Similarly, our method could be easily adapted to event detection in other noisy signals, such as the fluorescence of new voltage probes^47^ or even intracellular patch- and sharp-electrode recordings of super- and sub-threshold neuronal activity.

## Methods

### Software

MATLAB implementation of the MLspike and autocalibration algorithms are available as Supplementary Software, and can also be found on the depository https://github.com/MLspike. See also our Supplementary Note 1 and the two demos in the code for guidance in using MLspike.

### Experimental preparations and recordings

*Surgical procedures*. All experimental protocols were approved by the Marseille Ethical Committee in Neuroscience (rats; approval #A10/01/13, official national registration #71-French Ministry of Research), or by the Animal Care and Experimentation Committee of the Institute of Experimental Medicine of the Hungarian Academy of Sciences (awake mice; approval #PEI/001/194-4/2014 and 29225/004/2012/KAB). All procedures complied with the Hungarian Act of Animal Care and Experimentation (1998; XXVIII, section 243/1998.), French and European regulations for animal research, as well as with the guidelines from the Society for Neuroscience. All experiments on anaesthetized mice were conduced according to National Institute of Health guidelines and were approved by the Janelia Farm Research Campus Institutional Animal Care and Use Committee and Institutional Biosafety Committee^13^.

OGB-1-AM recordings were performed on juvenile Wistar rats (P28-40) of either sex. Those were anaesthetized with Urethane (2 g kg^−1^ body weight). Body temperature was monitored and maintained at 37.5 °C with a heat controller and heating pad (CWE). A metal chamber was attached with dental cement to the exposed skull above the primary somatosensory cortex (2.5 mm posterior and 5.5 mm lateral to the bregma). A 3-mm-wide craniotomy was opened and the dura mater was carefully removed. The chamber was then filled with agarose (2% in artificial cerebrospinal fluid) and stabilized under a cover glass. The latter was applied such as to leave a narrow rostro-caudal gap along the most lateral side of the chamber, in order to allow access to the micropipette used for dye injection or for electrical recordings.

The surgical procedures and strains for the anaesthetized mice GCaMP5k, GCaMP6f and GCaMP6s V1 experiments are described in refs 13, 15.

GCaMP6f recordings in awake mice V1 were performed in male C57BI/6J mice (P70-80). The surgery procedure was performed under anaesthesia with a mixture of midazolam, fentanyl and medetomidine (5 mg, 0.05 mg and 0.5 mg kg^−1^ body weight successively). V1 was localized first anatomically (0.5 mm anterior and 1.5 mm lateral to the lambda suture) and then confirmed functionally by intrinsic optical imaging. The rest of the surgical procedure was as described for rats. To awaken the mice from anaesthesia for the imaging, they were given a mixture of nexodal, reventor and flumazenil (1.2, 2.5 and 2.5 mg kg^−1^ body weight successively). Mice were kept head restrained in the dark under the two-photon microscope for about 1 h.

*Slice preparation*. GIN mice (P10-P24) anaesthetized with isoflurane were decapitated; their brain was rapidly removed from the skull and placed in ice-cold artificial cerebrospinal fluid (ACSF). The ACSF solution consisted of (in mmol): NaCl 124, KCl 3.50, NaH_2_PO4 1.25, NaHCO_3_ 25, CaCl_2_ 2.00, MgCl_2_ 1.30, and dextrose 10, pH 7.4. ACSF was aerated with 95% O2/5% CO2 gas mixture. Coronal slices (400 μm) were cut using a tissue slicer (Leica VT 1200s, Leica Microsystem, Wetzlar, Germany). Slices were transferred to a recording chamber continuously superfused (12 ml min^−1^) with ACSF (32 °C).

*Labelling*. Oregon Green 488 (OGB-1-AM, Molecular Probes) was bulk loaded by following the procedure described in ref. 48. Briefly, a glass micropipette (tip diameter 2 μm) filled with the dye (containing 1 mM OGB-1-AM and between 50 and 300 mM sulforhodamine SR101, to allow identification of neurons and glia, was prepared as in ref. 48). It was introduced below the cover glass from the side penetrating the cortex laterally and advanced towards the centre of the barrel, 300 mm below the cortical surface. The dye was pressure-injected under two-photon visual control at 3–10 PSI for 1–2 min. After the dye was taken up, neurons were labelled in a region of 300 mm diameter, centred on the injection site. In the *in vitro* application, the pipette was introduced into the slice up to 220–260 μm in depth and the dye was pressure-loaded under visual observation of green and red fluorescence overlay for 10 min until the slice surface reached staining levels yielding a fluorescence at least 40 times that of the green channel baseline.

The labelling methods of the GCaMP5k, GCaMP6f and GCaMP6s experiments in anaesthetized mice V1 can be found in ref. 13.

In the awake mouse experiments, V1 neurons were labelled by injecting adenovirus GCaMP6f construct AAV1.Syn.GCaMP6f.WPRE.SV40 (Penn Vectore Core, Philadelphia, PA). The injecting glass micropipette (tip diameter ∼10 μm) was back filled with 0.5 ml vector solution (∼6 × 10^13^ particle per ml) then injected slowly (20 nl s^−1^ for first 50 nl then 2 nl s^−1^ for the remaining quantity) into the cortex, at a depth of 400 μm under the pia, into V1. A cranial window was implanted 2 weeks after the injection over the injection site as described in Surgical procedures section.

‘Two-photon imaging' was performed using one of the two following setups: (i) a fast 3D random access AOD-based two-photon laser scanning microscope (Femto3D-AO, Femtonics Ltd., Budapest) described in ref. 11. A laser beam at 810 nm for OGB imaging and at 875 nm for GCaMP6f imaging was provided by a Mai Tai eHP Laser (Spectra Physics). We used either a × 20 Olympus objective, N.A. 0.95 or a × 16 Nikon objective, N.A. 0.8. (ii) A custom-built microscope described in ref. 4. A laser beam at 800 nm was provided by a Mira laser (Coherent) pumped by a Verdi 10W laser (Coherent). Scanning was performed with 6-mm-large scanning mirrors mounted on galvanometers (Cambridge Technology). Objectives: either a × 20 Olympus objective, N.A. 0.95 or a × 40 Zeiss objective, N.A. 0.8. In both setups, fluorescent light was separated from excitation light using custom-ordered dichroic filters and collected by a GaAsP photomultiplier (PMT) for the green calcium fluorescence and a multi-alkali-PMT for the red sulforhodamine fluorescence.

*Stimulation*. In the *in vitro* experiment, cells were stimulated using a tungsten electrode placed in the stratum radiatum of CA1 400 μm away towards the CA3 region from the imaged area. Ten stimulus pulses of 100 μA amplitude were applied at 1 Hz with 50 μs pulse width at using a stimulus isolator (WPI A365).

In the anaesthetized rat experiments, activity was recorded in the absence of a stimulus. For imaging and stimulation in anaesthetized mice, see ref. 13.

In the experiments performed on awake head-restrained mouse, a visual stimulus was delivered during data acquisition, in form of drifting gratings (spatial frequency: 0.25cyc/°, eight possible orientations). Those appeared after 2 s of dark screen, drifted for 5 s at 1 cyc s^−1^, stopped for 1 s, and were then replaced by the dark screen. For details see ref. 11.

The stimulation delivered during the GCaMP5k, GCaMP6f and GCaMP6s experiments in anaesthetized mice V1 can be found in ref. 13.

*Electrophysiological recordings*. After the preselection of neurons showing activity based on the bolus-loaded OGB1-AM, cell-attached (*in vivo*) or patch (*in vitro*) recordings were started on visually targeted neurons using borosilicate microelectrodes (6.1–8.5 MΩ) filled with ACSF containing 100 μM SR-101 (Life Technologies) for optimal visualization (overshadowing the glial cells in the red channel in Fig. 7a). When patching, the dye also served to check membrane integrity. Electrical recordings were made (Multiclamp 700B, Digidata1440, Molecular Devices) simultaneously with imaging. During *in vitro* recordings, temperature was kept at 32 °C (Supertech In-Line Heater, Supertech).

Table 1 summarizes type, origin and amount of the recorded data.

### Simple physiological model and reparametrization

Our model equations for OGB1 use equations given in refs 24, 32, 49 and reparameterize them so as to decrease the total number of parameters and use final parameters whose effects on the final dynamics are more intuitive.

The model input is a spike train 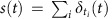, that is, a set of Dirac functions placed at spike times *t*_*i*_ distributed following a Poisson statistics of mean rate *λ*.

Free-calcium [Ca^2+^]_i_ evolution and fluorescence *F* measure are described in ref. 49 as


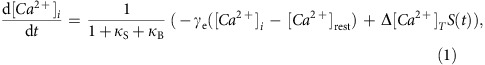



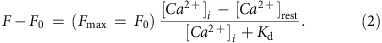


The different parameters are: [Ca^2+^]_rest_ the free-calcium concentration at rest; and *κ*_S_ and *κ*_B_ the calcium binding ratios, respectively, of endogenous calcium buffers and of the dye, with *κ*_S_ being constant, and *κ*_B_ being dependent both on the dye concentration and on [Ca^2+^]_*i*_ itself. However, in order to limit the total number of parameters, we simplify the model by ignoring the buffering capacity of the calcium indicator that results in slowed transient decays^32^; this means that *κ*_B_ is assumed to be constant. *γ*_e_ is the calcium extrusion rate; and [Ca^2+^]_*T*_ the calcium intracellular increase caused by one action potential (AP). *F*_0_ and *F*_max_ the fluorescence levels at rest and when the dye is saturated, respectively. *K*_d_ the dissociation constant of the dye.

To reparameterize these equations, we first introduce a ‘normalized intracellular calcium concentration' (at rest *c*=0, and upon the emission of one AP *c*=1):


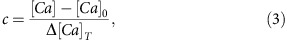


and a decay time constant parameter:


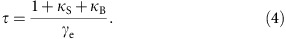


The calcium evolution equation (1) now becomes


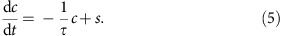


Similarly, we introduce a transient amplitude *A* and a saturation parameters *γ*:


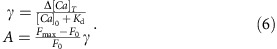


Note that *γ* is the inverse of the number of spikes for which the dye reaches half saturation. We can now replace the measure equation (2) with


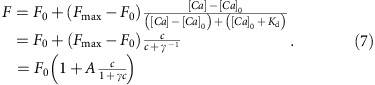


We also introduce, instead of the fix baseline *F*_0_, a drifting baseline *B*(*t*). This yields the model equations:


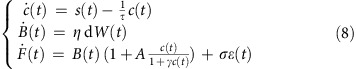


The ‘evolution noise' d*W(t)* denotes a Brownian motion and the ‘measure noise' *ɛ*(*t*) is white.

A major advantage of the reparameterization is to reduce the total number of parameters, which had redundant effects on the original model dynamics. Thus, our OGB model now has only six parameters: *A*, the relative fluorescence increase for one spike; *τ*, the calcium decay time constant; *γ*, a ‘saturation' parameter; *σ*, the amplitude of the expected measure noise; *η* the baseline drift amplitude; and *λ*, the rate of the Poisson spike train.

When *γ*=0, and *η*=0 (that is, *B*(*t*)=constant=*F*_0_), the model becomes linear and equivalent to a simple convolution


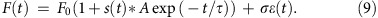


### Physiological models for GECI probes and reparameterization

In the case of GECIs, three different models were assessed. These three models are compared in Supplementary Fig. 8. The results displayed in Figs 5 and 6 use the first model, slightly modified by introducing a fixed delay (20 ms for GCaMP6s and 10 ms for GCaMP6f) between a spike and the (immediate) rise of the single exponential transient.

The first and largest difference between genetically engineered and organic calcium sensors is the supra-linear behaviour of the fluorescence response function to calcium. In the first model, we followed^15^, that is, fitted this function with a cubic polynomial:


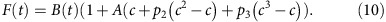


where *A* is the unitary fluorescence transient upon emission of a single spike (that is, when *c*=*c*^2^=*c*^3^=1). The Ca^2+^ and baseline evolution equations were kept unchanged.

In the second and third model (see Supplementary Methods for details), the supra-linear behaviour was modelled in a more physiological manner, by considering a cooperative binding of Ca^2+^ to the sensor^49^ (this introduced the Hill exponent parameter *n*, and the normalized Ca^2+^ concentration at rest *c*_0_, but the latter could be set to zero for our data), as well as dye saturation. In the second model, the measure function was thus replaced by


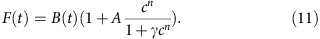


To account for the finite rise time of GECIs, in the third model we also introduced a rise time *τ*_on_ governing a non-immediate Ca^2+^ binding to the sensor. This increased the state dimension to 3, as the evolution of the fraction of probe bound to Ca^2+^, was now uncoupled from Ca^2+^ evolution.

The slower rise time is due to a slower calcium binding to the indicator, and the supralinear behaviour is due to the cooperative binding of more than one calcium ions to one indicator protein^49^. The full kinetics of the binding process should be taken into account then:


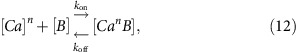



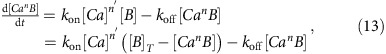


where [*B*] and [*Ca*^*n*^*B*] represent, respectively, the indicator free and bound to calcium, and [*B*]_*T*_=[*B*] + [*Ca*^*n*^*B*] is the total concentration of indicator; *k*_on_ and *k*_off_ are the association and dissociation rates (note that *K*_d_=*k*_off_ / *k*_on_); *n* is the number of binding sites per protein and *n'* is the Hill parameter: the true dynamics in (13) are best represented with a value of *n'* that does not necessarily match *n* but has to be determined empirically; however, for convenience, we will drop the ' sign in the following. Thus the evolution of calcium and bound indicator concentrations must be dissociated in two distinct terms, while the fluorescence measure (2) is replaced by


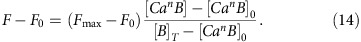


These new equations introduce a significant number of new parameters. To keep this number reasonable, we continue to ignore the buffering capacity of the calcium indicator that results in slowed transient decays, that is, we keep *κ*_B_ constant in equation (1), which can therefore still be rewritten as in equation (5); this is true in particular if the buffering of the dye is small (*κ*_B_<<(1+ *κ*_S_)); if calcium buffering by the dye is non-negligible, at least two additional parameter values would be needed.

We introduce the following normalized concentration of bound calcium indicator:


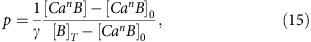


where the saturation parameter *γ* is updated as 
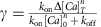
. Similarly to *c*, *p*=0 at rest and *P*≈1 for one spike, however contrary to *c*, *p* is upper-bounded by 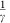, its saturation level.

We also introduce two new parameters: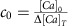 is the normalized level of baseline calcium concentration and 
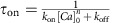
 is the binding time constant when calcium is at baseline.

We obtain


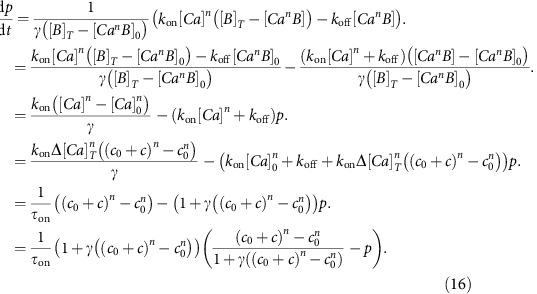


(the step between the second and third lines used that, at rest, we have 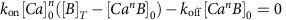).

The evolution and measure equations altogether can be written as


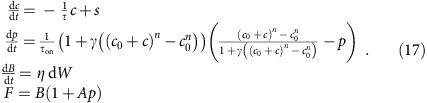


Note that they effectively reduce to equation (8) (that is, the second and fourth lines in (17) reduce to equation (7)) when *τ*_on_=0 and *n*=1.

### Time discretization and probability details

The model is discretized at the signal's temporal resolution *t* (below, *t* will be used for discrete time indices rather than continuous time). We will note the input as *n*_*t*_ (number of spikes between time *t*−*1* and *t*), the hidden state *x*_*t*_ (=*c*_*t*_ in the simplest model where baseline is constant and known, =(*c*_*t*_,*B*_*t*_) when baseline fluctuates, =(*c*_*t*_,*p*_*t*_,*B*_*t*_) when a rise time was introduced) and the measure *y*_*t*_ := *F*_*t*_.

We detail here this discretization and the full derivations of probability distributions *p*(*x*_*t*_*|x*_*t-1*_) and *p*(*y*_*t*_*|x*_*t*_) in the case of the simpler physiological model. The model equations become:


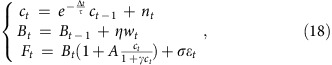


Random variable *n*_*t*_ follows an exponential law with parameter *λΔt*:


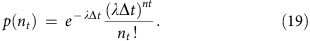


The other probability relations defined implicitly in the system (*w*_*t*_ and *ɛ*_*t*_ are independent Gaussian variables with mean zero and variance one) are


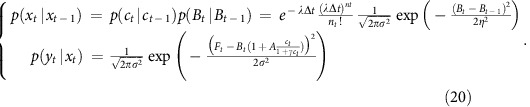


Note that the first line of the equation is a simplification for the more rigorous but complicate formula


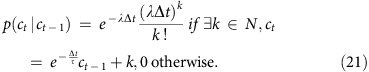


The last probability needed to fully describe the model is *p*(*x*_*1*_)= *p*(*c*_1_)p(*B*_1_). It is the *a priori* probability of the hidden state, in absence of any measurement. In practice, we used a uniform distribution for both *c*_1_ and *B*_1_.

Regarding *c*_1_, indeed we found that when the true spiking rate was not known, a uniform probability was better than a distribution determined mathematically based on the value of *a priori* spiking rate, because if that value was not correct, errors were increased. If the true spiking rate is known however, the following *a priori* can be used: one can observe that *c*_1_ is a weighted sum of Poisson random variables:


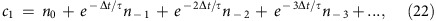


Its probability distribution can thus not only be computed exactly with iterative convolutions but is also well-approximated with a truncated normal distribution:


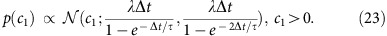


### Spike extraction algorithm

Let *T* be the number of time instants. We determined the best *x*=(*x*_1_,…,*x*_*T*_) that maximizes the posterior probability *p(x*_*1*_*,…,x*_*T*_*|y*_*1*_*,…,y*_*T*_), and hence obtain the best spike train *n*=*(n*_*2*_*,…,n*_*T*_), by using a dynamic programming algorithm, more precisely, a version of the Viterbi algorithm^33^. This approach relies on the following recursion of maximizations:


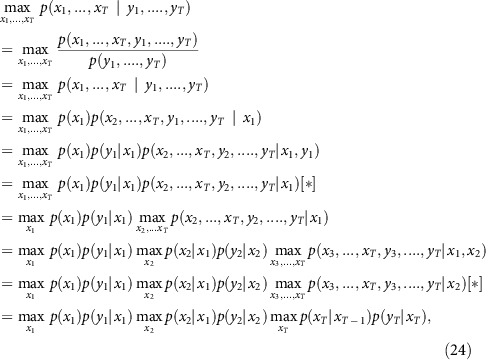


(steps marked with a star [] use the fact that both *y*_*t*_ and *x*_*t*−1_ are independent from (*x*_*t*+1,_ *y*_*t*+1,…,_ *x*_*T*,_ *y*_*T*_) conditionally to *x*_*t*_).

In other words, we can iteratively estimate a conditional ‘best probability' *m*_*t*_(*x*_*t*_):


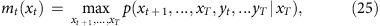


for decreasing values of *t*, starting with *t*=*T*. For each value of *x*_*t*_, the chain *x*_*t*+1_,…,*x*_*T*_ is the ‘best trajectory' starting from *x*_*t*_, as illustrated in Fig. 1b and the Supplementary Movie 1. In the general case where drifts are estimated, *m*_*t*_*(x*_*t*_) is a function defined over a 2D space (the set of all possible values for *(c*_*t*_*,B*_*t*_)), and can thus be easily encoded into a 2D array by using appropriate sampling values for *c*_*t*_and *B*_*t*_. This way of encoding probabilities is the basis of histogram filters (ref. 50).

At *t=T* we have *m*_*T*_(*x*_*T*_)=*p*(*y*_*T*_|*x*_*T*_), and for every *1≤t≤T−1:*


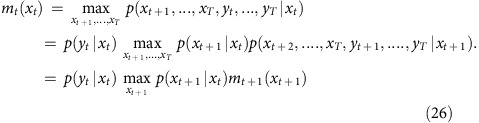


This iterative calculation of the conditional probabilities *m*_*t*_(*x*_*t*_) is illustrated in Fig. 1b (top and middle) and Supplementary Movie 1, in the simplified case where the baseline is known and constant, so *x*_*t*_ identifies with *c*_*t*_. Practically, for each value of *x*_*t*_, we store in memory the conditional probability *m*_*t*_(*x*_*t*_), and the best transition *x*_*t*_→ *x*_*t*+1_ (the arrows in the second part of the Supplementary Movie). But the full best trajectories *x*_*t*_,…., *x*_*T*_ do not need to be stored: only a single forward ‘collecting' sweep is performed at the end to determine 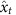 for increasing values of *t*, starting from 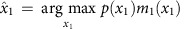 (Fig. 1b, bottom row).

### Implementing the discretization of the state space

To store in memory the conditional probability *m*_*t*_(*x*_*t*_), the state space needs to be discretized. However, when recursively computing *m*_*t*_(*x*_*t*_) (with *x*_*t*_ on the discretization grid), the *x*_*t+1*_ that realizes 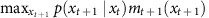 will typically fall outside of the discretization grid. Approximating to a value on the grid could lead to important estimation errors, unless the discretization grid is extremely dense, implying unreasonable calculation times and memory usage. Rather, we allow arbitrary values for *x*_*t+1*_, and interpolate to obtain the value of 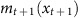.

Besides, not all possible values for *x*_*t*+1_ need to be considered but only a few. The maximization can be performed successively over different state variables, thanks to the independence of *B*_*t*_ and *c*_*t*_ evolutions:


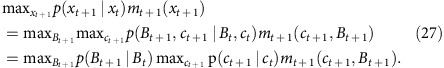


For maximization over calcium values, only discrete values of *c*_*t*+1_ corresponding to 0, 1, 2 or 3 spikes (we set a limit to three spikes per time bin) are allowed since evolution noise is absent:


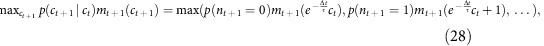


where all the 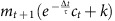 are obtained by interpolation: for all values of *c*_*t*_ on the grid, the vector of this quantity can then be interpolated from the vector of all *m*_*t*+1_(*c*_*t*+1_) through a single matrix multiplication, the interpolation matrix being precomputed before the recursion. With this respect, we found that a spline interpolation resulted on average in less error than a linear interpolation when coarsening the grid discretization to a point leading to estimation errors. It shall be noted also that these interpolation (of 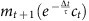, 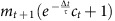 and so on, with *c*_*t*_ lying on the discretization grid) can be obtained by a simple matrix multiplication (applied to the *m*_*t*+1_(*c*_*t*+1_) vector), and that the interpolation matrix can be precomputed.

The maximization over baseline fluctuations is performed as follows: for each (*B*_*t*_,*c*_*t*_) on the discretization grid we need to find the *B*_*t*+1_that maximizes a certain function *f*_*Bt*,*ct*_(*B*_*t+*1_). First, the optimal value of *B*_*t*+1_ ‘lying on the discretization grid' is determined (and only a subset of the grid is considered, typically the five values centered on *B*_*t*_). Then a quadratic interpolation of *f* is computed using three local values as interpolating points, and minimized analytically, in order to yield an optimal value of *B*_*t*+1_ that does not have to lie on the grid. This quadratic interpolation is also obtained by a matrix multiplication, with the matrix being precomputed.

In our more detailed physiological model used for GECIs, we have introduced an additional state variable, *p*_*t*_, the normalized concentration of indicator bound to calcium. It shall be noted that this variable follows a deterministic evolution, therefore its introduction in equation (17) will only involve an additional interpolation for determining values with *p*_*t*_ on the discretization grid from values with *p*_*t*+1_ on the discretization grid, rather than an additional maximization. More specifically, we write


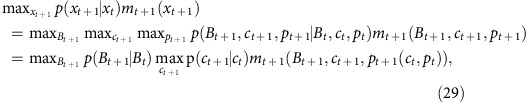


where 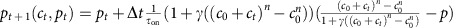, that is, *p*_*t*+1_ is a deterministic function of *c*_*t*_ and *p*_*t*_.

During the final forward sweep, the estimated *x*_*t*_ values are not restricted to lie on the discretization grid either. To change them from *x*_*t*_ to the next estimate *x*_*t*+1_ then, the number of spikes in the corresponding time bin is chosen based on the closest point on the grid, while the optimal baseline change is obtained by interpolating from the closest points on the grid.

Taken together, these techniques allow minimizing computation time by keeping the discretization grid relatively coarse (typically, we use 100 calcium values and 100 baseline values, but these number can in most cases be reduced to 30 without generating estimation errors), and by limiting the maximization search to a small number of tested values.

### Returning spike probabilities or samples instead of a unique MAP spike train

The algorithm can be modified to return spike probabilities in each time bin instead of a unique spike train, or a set of spike trains sampled according to the posterior probability.

To return ‘spike probabilities', we compute *p*(*x*_*t*_|*y*_1_,….,*y*_*t*_) and *p*(*y*_*t*_,…*y*_*T*_|*x*_*t*_) instead of 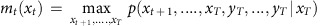, iteratively as


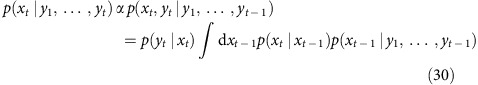


and


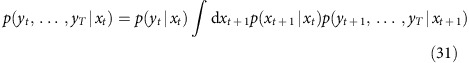


The expected number of spikes at time *t* is then obtained as


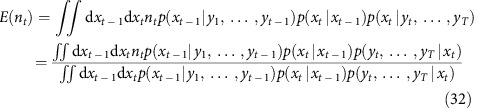


To return ‘sample spike trains' (and in fact, samples of the full calcium and baseline fluorescence dynamics) sampled according to the posterior distribution *p*(*x*|*y*), we first compute *p*(*y*_*t*_,…,*y*_*T*_|*x*_*t*_) iteratively as above.

Then arbitrary number of spike trains can be generated: they are initiated by drawing *x*_1_ according to


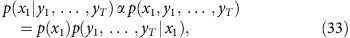


and iteratively drawing *x*_*t*_ according to


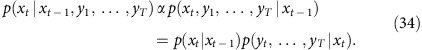


As for earlier MAP estimations, it is noteworthy that the abovementioned probability updates for one step in time can be decomposed into two sub-computations. For example, we have


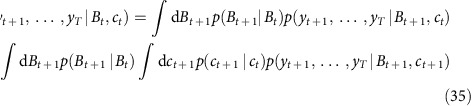


Two successive computations appear indeed. The first of them is actually a discrete sum:


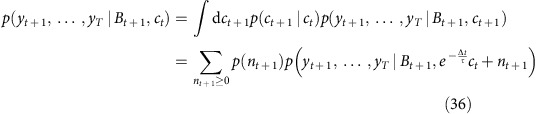


As earlier, this sum, which itself involves interpolations of 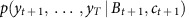, can all be obtained by a single matrix multiplication (the inner variable being *c*_*t*+1_), and the matrix can be precomputed.

The second computations is a continuous sum:


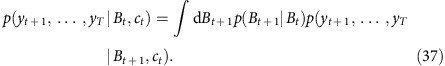


This sum can also be obtained by a single matrix multiplication applied to 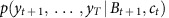 (the inner variable being this time *B*_*t*+1_).

### Autocalibration algorithm

Accurate estimations require accurately setting the six model parameters (for details on how each of them influences estimation quality, see Supplementary Methods). It would be tempting to estimate both the spike train and the parameters altogether by maximizing the likelihood. However, proceeding in this way not only proved to be computationally expensive but—more important—also led to less accurate spike train estimates than a more heuristic approach to estimate parameters *A*, *τ* and *σ* (see the dedicated paragraph in Discussion).

*Autocalibration of σ*. Parameter *σ* was estimated by computing the RMS of the fluorescence signals filtered between 3 and 20 Hz, and multiplying this quantity by a corrective factor. Indeed, our model considers fluorescence signals as the sum of calcium-related signals (possibly modulated by the baseline drifts) and of a white noise with s.d. *σ*.

It is possible to consider that the calcium-related part of the signals contributes significantly less to the high-frequency content of the signals (for example, above 3 Hz, see Fig. 3d) than the noise, so it appears justified to calculate the s.d. over high-pass filtered signals, and afterwards multiply it by a corrective term. The highest frequencies were also eliminated in this calculation, because the noise present in our data is actually not purely white (see the spectra in Fig. 5a), implying that it might not be accurate to use signal power calculated in the highest frequencies to estimate the noise level expected in the crucial band ∼1 Hz.

The corrective factor was determined such that when the method is applied to a white noise signal, the estimated *σ* value corresponds to its true standard deviation. However, at low SNR, estimating *σ* to this ‘correct' value can lead to an excessive number of misses, the algorithm ‘not trusting the data enough to assign spikes'. Therefore for the OGB and GCaMP6f data set, we slightly biased this factor (multiplying it by 0.7), to force *σ* to be underestimated. This resulted in a more equilibrated number of misses and false detections.

*Autocalibration of A* and *τ*. As shown in Fig. 4a (see also Supplementary Fig. 5), the autocalibration of *A* takes advantage of the discrete nature of spikes, namely that calcium transient amplitudes can take only a fix set of values depending on whether they are caused by 1, 2, 3 and so on spikes. Noise obviously increases the variability, but it is possible to obtain histograms of transient amplitudes that show several peaks corresponding to different numbers of spikes.

Parameters *A* and *τ* are estimated together according to the steps detailed below (see also Supplementary Fig. 5a).

First, the spike estimation algorithm is modified such that the estimated input *s(t)* is not any more a spike train with unitary events, but a set of ‘calcium events' of arbitrary amplitudes (although a minimal amplitude of *A*_min_ is imposed, for example, =4% for OGB).

This is achieved by modifying equation (19) as follows:


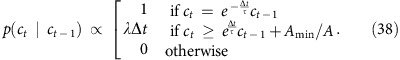


For this estimation, we use *A*=10%, *τ*=0.8s. *σ* is autocalibrated as explained in the previous section. Standard, experience-inspired default values are used for the nonlinearity parameters, drift parameter and ‘calcium event rate' *λ*.

Next, the amplitude of single spike transients is best estimated from isolated calcium transients of moderate amplitude. Therefore calcium events that are either too close (<1 s) to another event, or of amplitude *F/F* >25% are excluded. The predicted calcium signals for those excluded events are then subtracted from the original signal, yielding modified calcium signals containing only the ‘good' events. Individual event amplitudes and the value of *τ* are then re-estimated so as to maximize the fit to these new signals.

At this point, a histogram of all event amplitudes is constructed (Supplementary Fig. 5a). It is first smoothed, yielding *x*_1_. Thereafter, peaks are enhanced by dividing *x*_*1*_ by a low-passed version of itself, *x*_2_. A cost function *x*_*3*_ is then defined as *x*_3_(*A*)=*x*_2_(*A*)+*x*_2_(2*A*)/2 over a bounded range [*A*_min_=4%, *A*_max_=10%] (note that ‘*x*_2_(2*A*)' is a simplified view: in general 2A is replaced by the actual amplitude of a two-spikes transient taking into account nonlinearities). A first estimate of *A* is chosen as the value that maximizes *x*_3_ (the green star in Supplementary Fig. 5a). This estimate is used to assign a number of spikes to each individual event (black separation lines and printed spikes numbers): the separations between *k* and *k*+1 spikes are set at (*k*+0.3)*A.

Finally, a standard calibration routine is used to estimate final values of *A* and *τ* by maximizing the fit to the modified calcium signals.

Spike estimation results based on autocalibration values of *σ*, *A* and *τ* are shown for the same data set in Supplementary Fig. 5b.

Naturally, some of the parameter values given above for OGB (for example, *A*_min_ and *A*_max_) had other values for GCaMP6s and for GCaMP6f due to different dynamic ranges for *A* and *τ*. As a final note, several parts of the autocalibration algorithm being based on somewhat intuitive heuristics, large room for ameliorations is expected, notably through a more rigorous formulation.

*Other parameters (currently not autocalibrated)*. Rise time *τ*_on_ should be easy to autocalibrate in many different situations, since spikes can be reliably detected first without a rise time, and then be used to autocalibrate *τ*_on_.

We did not need to autoestimate the baseline drift parameter *η*, as our optimized estimations (Fig. 5b) showed that the optimal value for *η* varied only little between different sessions and cells. We thus assigned *η* a fixed value. *A priori* expected spike rate *λ* and noise level *σ* are linked in their effect on the estimations, as illustrated at the beginning of this section. Because of their redundancy, we could fix *λ* to 0.1 (and autocalibrate *σ*).

Nonlinear parameters (that is, saturation *γ*, Hill exponent *n* or polynomial coefficient *p*_2_ and *p*_3_) appear more difficult to estimate from calcium signals alone, as they mostly modulate calcium during periods of high spiking rates, where it is more difficult to distinguish the responses to individual spikes. We thus expect autoestimation to be successful only at very high SNR. Otherwise, using a fixed value is preferable: we used the average over all neurons that were calibrated with simultaneous patch recordings (*γ*=0.1 for OGB, [*p*_2_,*p*_3_]=[0.73, −0.05] and [0.55, 0.03] respectively for GCaMP6s and GCaMP6f).

### Details on simulations and real data estimations

A summary of details on simulations and estimations shown in this study, such as parameter values, settings and so on, is provided in our Supplementary Table 1.

### Simulations

Simulated spike trains generally consisted in Poissonian trains (Figs 2a,b and 3a–c, and Supplementary Figs 1–4). However, in the ‘autocalibration' simulations (Fig. 4b,c), more realistic trains were generated, which could include spike bursts: bursty events were generated at a fixed rate, then a number of spikes was randomly assigned to each event according to an exponential distribution (average 1 spike per event, some events had 0 spikes), finally inter-spike intervals within these events were drawn from a Gaussian distribution with 10 ms mean.

For all simulations except for those used to test the autocalibration, the true parameter values for *A, τ* and *γ* were given to the algorithm, while other parameters were optimized separately for each different condition so as to minimize ER: *σ* and *η* in the case of MLspike, and parameters playing equivalent roles for the Peeling (see the dedicated section below). Also, in the case of ‘no drift' simulations, the constant baseline value was either provided to the algorithm (Supplementary Fig. 2), or not (Fig. 2 and Supplementary Fig. 1). In the latter case, this constant value had to be estimated, which is one of MLspike*'s* capabilities, but not of Peeling.

### Real data

When running ‘optimized' MLspike*'s* estimations, physiological parameters (*A*, *τ* and if applicable *γ*, *n*, *p*_2_, *p*_3,_ *τ*_on_) were calibrated using the simultaneous electrical recordings (that is, were optimized such as to best predict the calcium signals from the recorded spikes). Other parameters (*σ* and *η*) were optimized such as to best estimate spikes from the recorded calcium signals (for some OGB neurons recorded with the AOD system, different recording settings had been used in different acquisitions: in such cases these other parameters—but not the physiological ones—were optimized separately for each setting).

In ‘autocalibration' MLspike estimations, parameters *A*, *τ* and *σ* were estimated from the data themselves (for the multi-session neurons mentioned previously, all trials were pooled together for the estimation of *A* and *τ*, while *σ* was estimated for each session independently). Other parameters were assigned some fixed values: the physiological parameter(s) (if applicable *γ*, *n*, *p*_2_, *p*_3,_ *τ*_on_) were assigned average calibrated values (see table in Supplementary Fig. 6a), and the value of drift parameter *η* was found heuristically (autocalibration was not performed for GCaMP5k and the two awake GCaMP6f cells because of lacking single-spike resolution).

We also compared MLspike ‘autocalibration' estimations with estimations with all parameters fixed to the average calibration values obtained from our data (Fig. 5d): physiological parameter(s) were assigned the average calibrated value (see table in Supplementary Fig. 6a), and parameters *σ* and *η* were found heuristically.

Finally, in the case of OGB, we also performed the estimations using fixed parameter values from ref. 10 (Fig. 5d, left). This study reports calcium transients best being fitted by the sum of two exponentials, one with a fast, the second with a slower decay constant (*A*_1_=7.7%, *τ*_1_=56 ms, *A*_2_=3.1%, *τ*_2_=777 ms). Peeling has the ability to model transients with two such exponentials but does not model dye saturation effect (see also next section). MLspike, which currently assumes a single-exponential model, was used with the parameters for a single exponential that best fitted the sum of the two abovementioned ones (*A*=6.27%, *τ*=366 ms) and no saturation, for a fair comparison with Peeling. Despite these approximations MLspike performed better than Peeling (average ER of 29.2% (Fig. 5d, left, ‘fixed (liter.)') compared with 35.8% (Fig. 6b, two leftmost graphs, ‘Peeling'). When Peeling was run using the same approximation with a single exponential rather than two it performed even worse (38.2%—not shown).

### Other algorithms tested

*Peeling algorithm*. The Peeling algorithm^10^, similarly to MLspike, returns a unique estimated spike trains that accounts for the recorded fluorescence signal. It requires a certain number of physiological and algorithmic parameters to be set.

Regarding algorithmic parameters, preliminary testing of the algorithm on simulated and real data allowed us to determine which parameters could be kept fixed to their default value, and which are needed to be tuned depending on the quality of the data. We found three such parameters: the first one, *noiseSD* controls the expected level of noise, by scaling the values of two other parameters: schmittlow=1.75*noiseSD and *schmitthigh*=−*noiseSD*; note that in the simulations for Supplementary Fig. 2 these two parameters were optimized independently. Two other parameters, *slidwinsiz* and *maxbaseslope*, had to be tuned according to the level of baseline drifts in the signals. In all simulations these parameters were optimized independently for each conditions (Supplementary Figs 2 and 4), while on real data they were assigned fixed values found heuristically.

Regarding physiological parameters, all comparisons on simulated data involving Peeling were performed with known values of parameters *A* and *τ*, and assumed linearity of the indicator. Peeling has an option for performing nonlinear estimations that account for dye saturation; however, this option resulted in poor baseline drift estimations, even after we edited and improved the code, therefore all Peeling estimations even on real data were rather performed using the linear model. On our OGB data set, we used the ability of Peeling to model calcium transients with two exponentials; values from ref. 10 were used (*A*_1_=7.7%, *τ*_1_=56 ms, *A*_2_=3.1%, *τ*_2_=777 ms), and this resulted in slightly better estimation accuracies than with only one exponentials (average ER 35.8% compared with 38.2%, see the section above). In the case of GECIs estimations, using Peeling with our average calibrated values for parameters *A* and *τ* led to underestimating the amplitude of calcium responses to bursts of spikes, since Peeling does not model the dye supralinearity (by doing so we obtained an average ER of 36.7%, not shown). Rather, we increased *A* by replacing it by half of the response to two spikes: in that way, responses to one spike were slightly overestimated while responses to bursts of more than two spikes were still underestimated (this led to <ER>=32.1%, as shown in Fig. 6b).

Finally, to take into account the finite risetime in the case of GEGIs, for the precise temporal quantifications in Fig. 6, we applied the same correction to Peeling as to MLspike (see Methods section ‘Model') and *SMC* (see below). That is, we assumed a fixed delay (20 ms for GCaMP6s and 10 ms for GCaMP6f) between a spike and the (immediate) rise of its single exponential fluorescence transient (that is, estimated spike times were moved backward by this delay).

*Sequential Monte-Carlo, Constrained Deconvolution and Markov Chain Monte Carlo*. We compared our real data estimations also to three algorithms published by the Paninsky group^24^,^26^,^27^. These algorithms have in common that they estimate model parameters directly from the data, either in a direct or iterative fashion, thus requiring no or little parameter tuning. They all return an estimated spiking rate (or spiking probability; up to a scaling factor in the case of CD) at each time point of the original fluorescence signal, but the MCMC algorithm does this by generating a number of spike trains theoretically sampled from the posterior distribution that can be directly used, for example, for error quantification. Their underlying dynamic models are simpler than the one used for MLspike, as they do not include dye saturation for CD and MCMC (SMC does include it), and, more importantly, do not include baseline fluorescence fluctuations (SMC includes noise in the calcium evolution that can account for part but unfortunately not all of spike-unrelated fluctuations in the signals). The CD algorithm thus relies on the same model as MCMC, but it also entails simplifications that greatly increase computation speed at the expenses of accuracy^27^; in fact MCMC estimations are initialized with the result of CD estimations similarly, SMC estimations are initialized with the result of the ‘fast_oopsi' algorithm^25^.

On our data sets, we observed that the lack of baseline fluctuations in the models could lead to important errors, for example with large inaccurate spiking activity being estimated where the baseline was higher. We therefore improved the estimations by detrending the signals before applying the algorithms: this increased estimation accuracies of the three algorithm; we also tried high-pass filtering the signals (having noticed that signals are high-pass filtered in ref. 24), but this proved less efficient than detrending. We further improved the accuracy of MCMC by imposing minimal values for parameter *A* (the same as for our own autocalibration algorithm, that is, OGB and GCaMP6s: 4% ΔF/F; GCaMP6f: 2.5%), as this prevented the algorithm to fit baseline drifts with transients of small amplitudes. Similarly to MLspike and Peeling, in the case of GECIs, we corrected SMC estimations with a fixed delay of 20 and 10 ms (for GCaMP6s and GCaMP6f, respectively). MCMC did not require such a correction because its estimations were run with an autoregressive model of order 2, which takes into account the finite rise time.

A specific advantage of our MLspike implementation is that autocalibration can be performed globally on many trials recorded from the same neuron. Although there is no conceptual that would prevent doing the same for SMC, CD and MCMC, the publicly available code does not do it. We therefore improved the code of CD and MCMC so as to estimate, for example, for MCMC, spikes from *n*>1 trials from the same neurons, a single value for transient amplitude and time constant(s) parameters, and *n* (one per trial) values for the baseline fluorescence and initial calcium concentration. This in fact improved overall estimation accuracy only very slightly, with improvements for some neurons but deteriorations for others: probably in the latter case neurons mismatcheing with the model (for example, baseline fluctuations) in some trials were misleading the global parameter estimation, therefore decreasing estimation accuracy in other trials.

If, as opposed to Peeling, we did not need to set parameters for the estimations, we did change a few default algorithmic parameters to increase the robustness of estimations (at the expense of speed). Namely, the number of EM iterations for SMC was increased to 6; numbers of burn-in and used samples for MCMC were both increased to 400 (for example, 800 sample spike trains were generated, and only the last 400 were kept). Finally, because of their probabilistic nature the SMC and MCMC algorithms yield slightly different results when being repeated on the same data; to ensure repeatability; we thus reinitialized the random number generator previously to each estimation.

### Quantification of estimation accuracy

*Error rate*. Once spike trains have been estimated, they need to be compared with the real simulated or electrically recorded spikes. We used the *F*_*1*_-score to define an ER, defined as the harmonic mean between sensitivity and precision^51^:


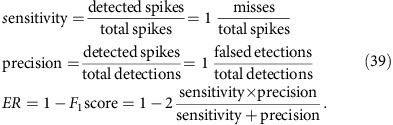


We consider a given spike detection correct when it matches a real spike with a temporal precision better than 0.5 s (smaller upper bounds for the acceptable temporal precision were also tested, see Fig. 6b–d). The estimated and real spikes were matched by computing distances using a simple metric over spike trains^52^ that assigns costs to spike insertions, deletions and shifts, and is calculated using a dynamic programming algorithm.

When quantifying the error for estimations in several trials from the same neurons, we counted together all the true/detected/missed/falsely detected spikes from different trials, in order to yield one single sensitivity, precision and ER value for this neuron (the alternative of computing one ER value per trial and averaging over trials yielded very similar results).

ER could be computed not only on the estimates returned by MLspike and Peeling, which both output a single spike train, but also of MCMC. This was done by counting together all the true/detected/missed/falsely detected spikes from all the sampled spike trains generated by the algorithm. This resulted in an average ER, noted <ER>, that reflected the average accuracy over this distribution of spike trains.

*Correlation*. To compare estimation accuracies to algorithms estimating spiking probabilities, we used correlation between the vectors of real spike counts after binning to 40 ms (or other if specified), and the estimated instantaneous spiking rates. These instantaneous spiking rates were either directly provided by the algorithm (MCMC, CD and *SMC*) or obtained by low-pass filtering estimated spike counts (MLspike and Peeling) with a kernel of 100 ms.

### Quantification of the noise level

*Noise level*. We quantified the noise level in the real data by taking the RMS of the difference between the measured fluorescence signals and those predicted by the electrically recorded spikes (using the calibrated parameter values). Before computing this RMS however, the signals were filtered between 0.1 and 3 Hz (in Supplementary Fig. 8d,e, we also show the result of other filterings, more optimal for specific probes). Then, this RMS was normalized by a quantification of the signal amplitude. In the case of the simulations or of the OGB data, using parameter *A* for this quantification led to satisfying properties of the noise level. However in the case of GECIs, noise levels calculated that way could become very high due to weak responses to single spikes (while, at the same time, leading to underestimating the strong responses to bursts). We therefore preferred normalizing by ‘*A*', the ‘average response to one spike', defined as half of the response to two spikes in the case of OGB, GCaMP6s and GCaMP6f, and 1/15 of the response to 15 spikes in the case of GCaMP5k. Note that *A*'*≤A* in the case of OGB due to saturation, and *A*'*⩾A* in the case of GECIs due to supralinearity.

*Calibration of the PMTs in order to estimate the photonic contribution to the noise*. In addition to the ‘noise level', we also display full spectra of the noise (normalized by A') next to example signals and estimations. It was even possible to determine which part of this noise corresponded to photonic noise by an independent calibration of the PMTs, where we measured photonic noise corresponding to different signal levels and at different PMT voltages.

Indeed, the variance of the photonic noise is proportional to the number of photons collected by the PMT: if *s* is a signal whose noise is purely photonic, we note as *N* the corresponding average number of photons collected per time bin and *a* the gain of the PMT:


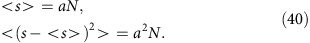


Therefore the gain can be estimated as 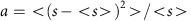.

However, this is true only when the variance in the signal is only due to photonic noise. Even when imaging steady signals from fluorescent beads, we cannot estimate *a* in this manner because their signals will always contain system noise as well, which is non-negligible compared with photonic noise.

Fortunately, system noise becomes negligible compared with photonic noise at high frequencies, for example, above 200 Hz. Thus, we imaged beads at high frame rate (for example, *f*_*s*_=1 kHz; we note *s*_*b*_ the obtained signals). Then we high-pass filtered these signals above *f*_*c*_=200 Hz (we note the result 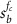). The variance of 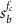
is now due purely to photonic noise, which we note: 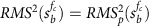. To relate this variance to the total photonic noise of original signal *s*_*b*_, we use the fact that the photonic noise is a white noise, and therefore has a flat spectrum homogeneously distributed between 0 and *f*_*s*_/2. Therefore, we have


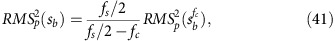


and the PMT gain could then be estimated as


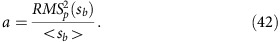


Then for any new signal *s* acquired at the same PMT voltage at a given frame rate *f*, the contribution of photonic noise to the total noise RMS is 
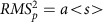
, and using the same argument as above of the flat spectrum of the photonic noise, its contribution inside a specific frequency band [*f*_1_ *f*_2_] is 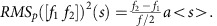


### Data availability

The GCaMP5 and GCaMP6 data used in this work are available at http://crcns.org/. All other data are available from the authors upon request.

## Additional information

**How to cite this article:** Deneux, T. *et al*. Accurate spike estimation from noisy calcium signals for ultrafast three-dimensional imaging of large neuronal populations *in vivo*. *Nat. Commun.* 7:12190 doi: 10.1038/ncomms12190 (2016).

## Supplementary Material

Supplementary Information Supplementary Figures 1-10, Supplementary Table 1, Supplementary Note 1 and Supplementary References

Supplementary Movie 1 Schematic explanation of MLspike algorithm. The first part, similar to Fig. 1b, presents the abstract concept of iteratively estimating the probabilities of 'best trajectories' originating from different calcium values. The second part shows more rigorously how the backward and forward sweeps are implemented, in particular using probability discretization and interpolations.

Supplementary Software MATLAB implementation of the MLspike and autocalibration algorithms are provided here. Eventual software upgrades will be available at https://github.com/MLspike

## Figures and Tables

**Table 1:** Data summary.

**Indicator**	**System (if not specified,** ***in vivo*** **anaesthetized)**	**Setup**	**Experiment location or shared data**	**#cells**	**Min #trials per cell**	**Max #trials per cell**	**Average trial length**	**Average time per cell**
OGB	Rat barrel cortex	AOD	CNRS, Marseille	13	4	75	25 s	9 min
OGB	Rat barrel cortex	galv.	Weizmann Institute	10	2	28	25 s	3.5 min
OGB	Mouse hippocampus, *in vitro*	AOD	CNRS, Marseille	1	10	10	25 s	4 min
GCaMP5k	Mouse visual cortex	galv.	refs 13,15	9	1	1	193 s	3 min
GCaMP6s	Mouse visual cortex	galv.	refs 13,15	9	1	4	216 s	8 min
GCaMP6f	Mouse visual cortex	galv.	refs 13,15	11	1	6	222 s	13 min
GCaMP6f	Mouse visual cortex, awake	AOD	IEM, Budapest	2	3	4	9 s	30 s

‘AOD' and ‘galv.': data acquired with microscope using acousto-optic and galvanometric scanning, respectively. OGB, Oregon Green BAPTA-1-AM.
