# Supplementary material for: Accurate spike estimation from noisy calcium signals for ultrafast three-dimensional imaging of large neuronal populations in vivo
Source: Nat Commun. 2016 Jul 19;7:12190. doi: 10.1038/ncomms12190 (PMC4960309; doi:10.1038/ncomms12190)
Supplement: Supplementary Information — Supplementary Figures 1-10, Supplementary Table 1, Supplementary Note 1 and Supplementary References [file ncomms12190-s1.pdf]

Supplementary Figures and Legends

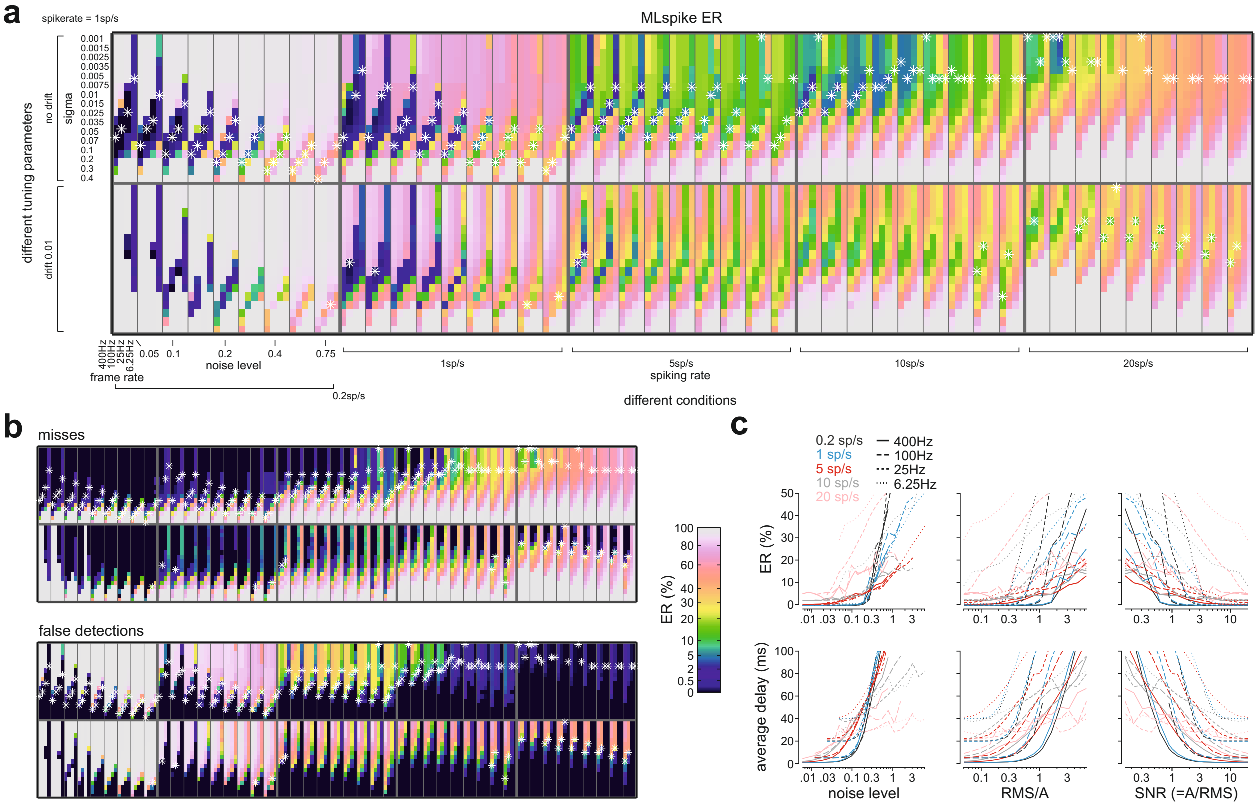

Supplementary Figure 1: **Parameter dependencies; temporal accuracy. Case 1: white noise, flat but unknown baseline.** Panel **a** expands all estimation errors for different conditions (columns: frame rate, noise level and spiking rate) and different *MLspike* parameter settings (rows: *sigma*, the expected RMS of noise in the data, and *drift*, the amount of allowed baseline drifts/fluctuations; parameter *spikerate* is fixed to 1sp/s). Optimal parameter settings are indicated with white stars and correspond to simulation results presented in Fig. 2b. Color codes for error rate (ER). Between 2 and 120 simulations were run for every data point, the exact number of repetitions being adjusted to the datapoint's relevance (e.g., only few repetitions were run for datapoints with very large ER). At lower noise levels, the performance of *MLspike* deteriorated only slowly when *sigma* moved away from its optimal value (note its range covering more than two orders of magnitude). At a given frame rate and noise level, "good parameter" ranges were similar up to spiking rates of 5sp/s, which means that estimation can be run without needing to know a priori the exact spiking rate; for 10sp/s and 20sp/s spiking rates however, the algorithm needed to be assigned to more consider signal variations as spikes rather than noise by setting smaller values for *sigma*. Somewhat surprisingly, in a few cases mostly limited to the highest spiking rates and sampling frequencies, estimation was slightly more accurate when the algorithm was allowed a small amount of baseline fluctuations, despite the actually flat baseline (bottom

part, *drift*=0.01). We speculate that the resulting additional flexibility allowed the algorithm to capture more spikes based on their sole onset. **b**: Same as (a), but displaying misses and false detections separately. **c**: Error rate and mean spike time error as a function of noise. Data are the same as in Fig. 2b, but plotted also using two other noise quantifications. Parameters were set to optimal values (white stars in (a),(b)). Note that when noise is quantified by "noise level" (RMS between 0.1Hz and 3Hz), the algorithm's performance becomes largely invariant with respect to frame rate. This underscores the fact that the fluorescence time series' relevant information is contained within the frequency range that covers the essential part of the individual action potential's fluorescence response spectrum (Fig. 3d).

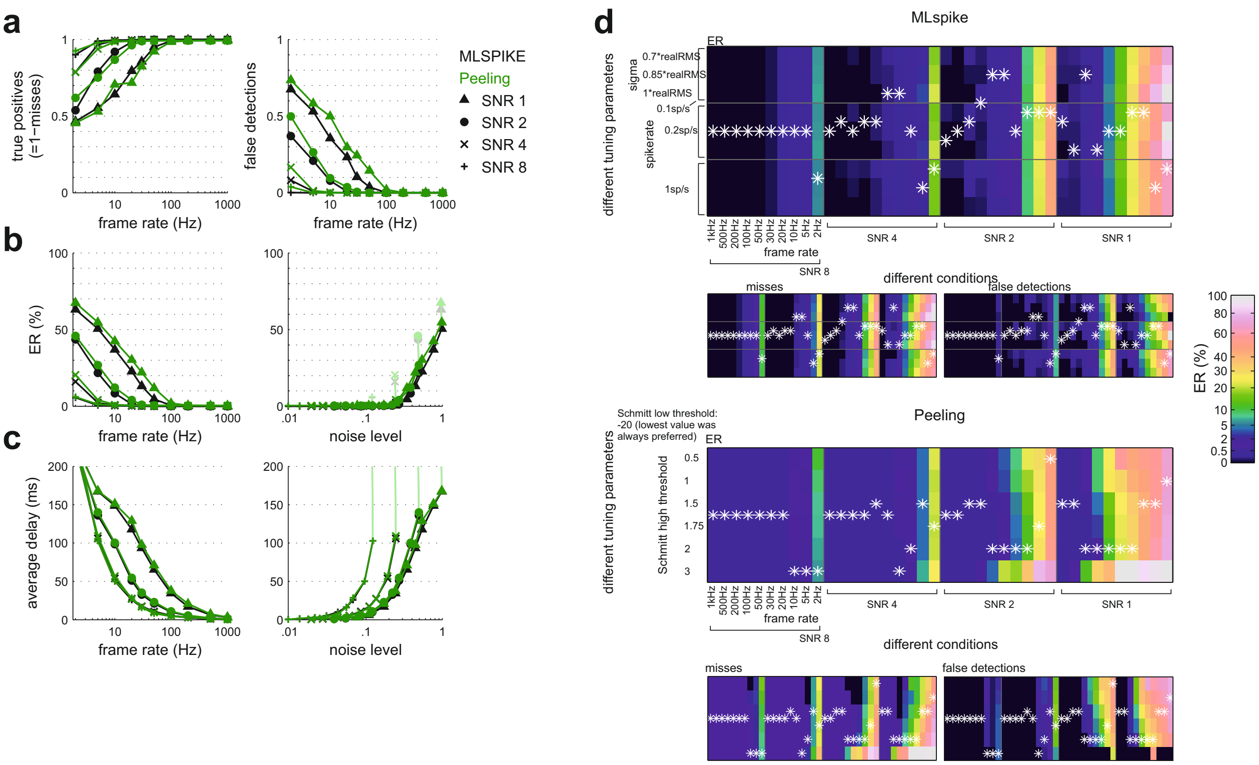

Supplementary Figure 2: **Comparison of *MLspike's* performance with that of the *Peeling* algorithm on white noise, flat and known baseline; parameter dependencies. a**: Reproduction of the simulations in Fig. 3 of[1], using Poissonian spike trains (spike rate = 0.2sp/s) at several (white) noise levels, quantified as SNR = A/RMS(noise) (The *Peeling* algorithm was run using the code available online – which indeed reproduced the published results[1]). **b**: Summarizing error rate as function of SNR (left) or noise level (right). When baseline was flat and its value known as was the case here and in[1], *MLspike* and *Peeling* performed much similarly. Yet, even in such restrictive (and in practice rare) conditions, *MLspike* slightly but consistently outperformed *Peeling* at lowest SNR (=1). Note that when plotting the error as a function of noise level, the points for different frame rates overlap, underscoring the adequacy of our noise level quantification (except for frame rates < 3Hz, at which noise level is ill-defined: 2Hz points shown in light green). **c**: Mean spike time error. Note that the temporal accuracy of data at low SNR is limited by the noise (the SNR=1,2 curves overlap when plotted as function of

noise level), while temporal accuracy at high SNR is limited by frame rate (the SNR=4,8 curves overlap when plotted as function of frame rate). **d**: Dependency of both *MLspike* and *Peeling* on its most important parameters (graphical conventions as in Supp. Fig. 1). Curves in (a-c) were obtained using optimal parameter settings (white stars in (d)).

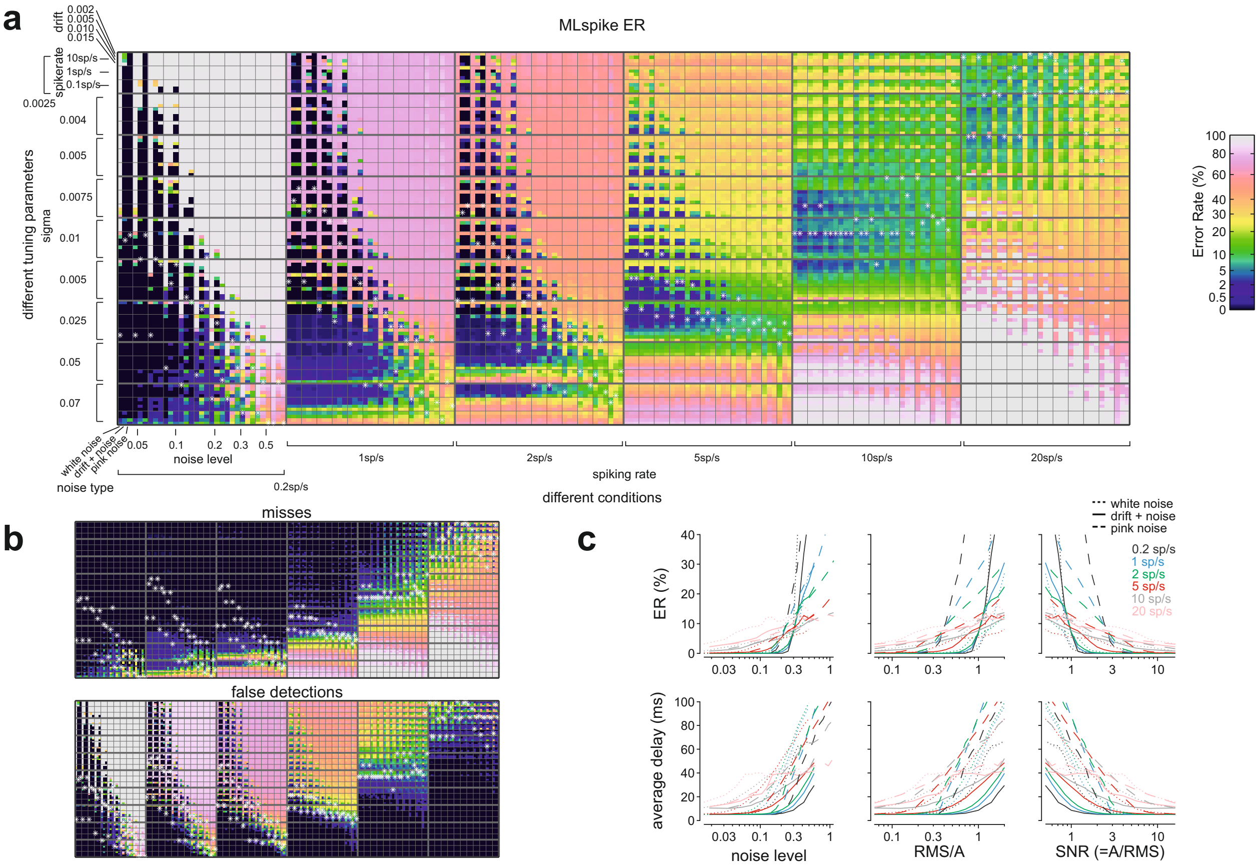

Supplementary Figure 3: **Parameter dependencies; temporal accuracy. Case 2: different types of noise.** As in Supp. Fig. 1, panel **a** shows all estimation errors for different conditions (columns: noise type, noise level and spiking rate) and different *MLspike* parameter settings (rows: *drift*, *spikerate* and *sigma, see* Supp. Fig. 1 for their meaning). It expands Fig. 3c. As expected, the most critical parameter was *sigma*, higher noise levels calling for larger values (note also its smaller range as compared to Supp. Fig. 1) in order to limit the number of false detections (b), therefore restricting the range of good parameter values. **b**: Same as (a), but displaying miss- and false detection rates separately. **c**: Error rate and spike time error. Same simulations as in Fig. 3c, but plotted using three different noise quantifications. Parameters were set to optimal values (white stars in (a),(b)). Once more, quantifying noise as "noise level" allows to strongly reducing *MLspike's* performance dependency on the type of noise. Frame rate: 100Hz in all panels.

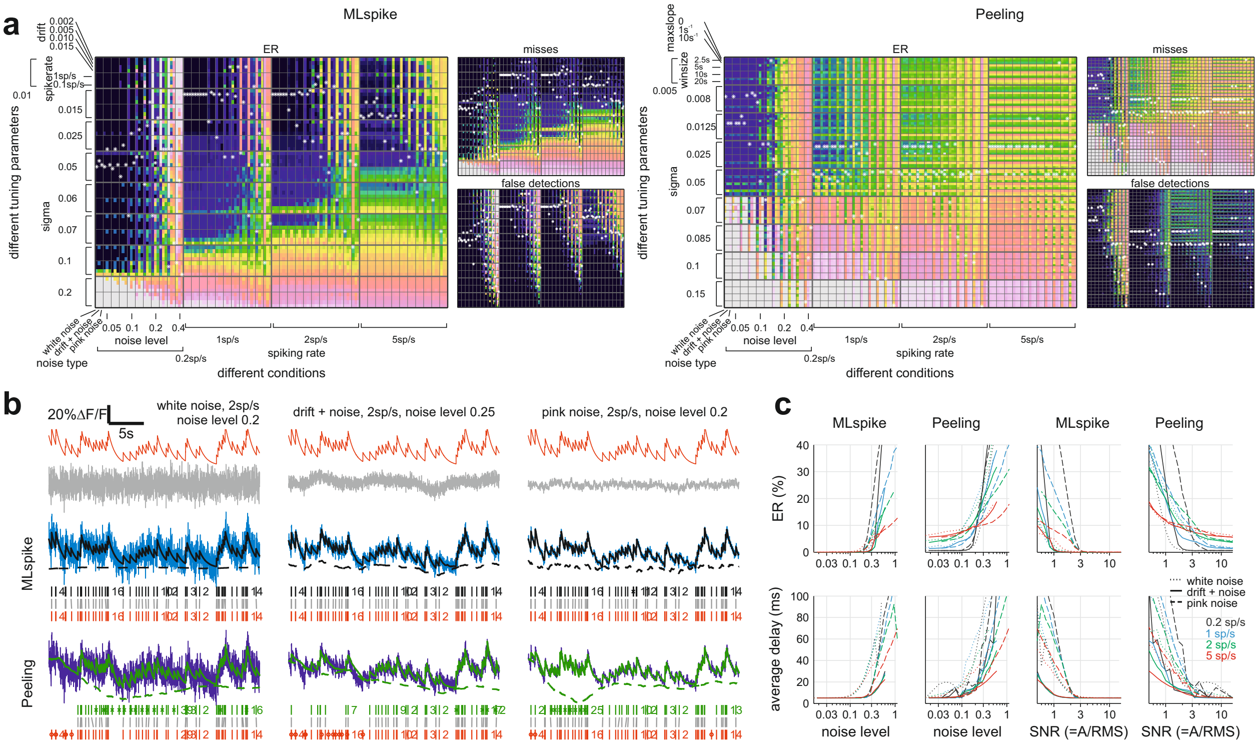

Supplementary Figure 4: **Performance comparison of *MLspike* with *Peeling* on different noise types; parameter dependencies.** These simulations are similar to those in Fig. 3c and S3, but without any calcium saturation (allowing to use the linear version of *Peeling*, which in our hands is more stable). Moreover, here, the baseline fluctuations have an *additive* rather than *multiplicative* effect, i.e., the signal increase for one spike is fixed to a constant value – as required by the *Peeling* algorithm. On the contrary, in all other simulations the signal increase was scaled by the baseline value. This is more rigorous but also more delicate to handle, in particular at high spiking rates where exact baseline estimation is difficult. **a**: Dependency on tuning parameters. **b**: Estimation on example traces; as in Fig. 3, blue traces (simulated fluorescence signals) are the sum of red (noise-free fluorescence signals) and grey (noise) traces. Three estimation examples (same graphical conventions as in Fig. 2,3) are displayed in black (*MLspike*) and green (*Peeling*). Their noise levels and ERs are marked by green/black circles in (c). Note that the difference in performance between the two algorithms is mostly due to a better ability of *MLspike* to discriminate between calcium transients due to spikes and baseline fluctuations. **c:** ER and the average delay between estimated and true spike times were plotted as a function of noise level and SNR. Frame rate = 100Hz in all panels.

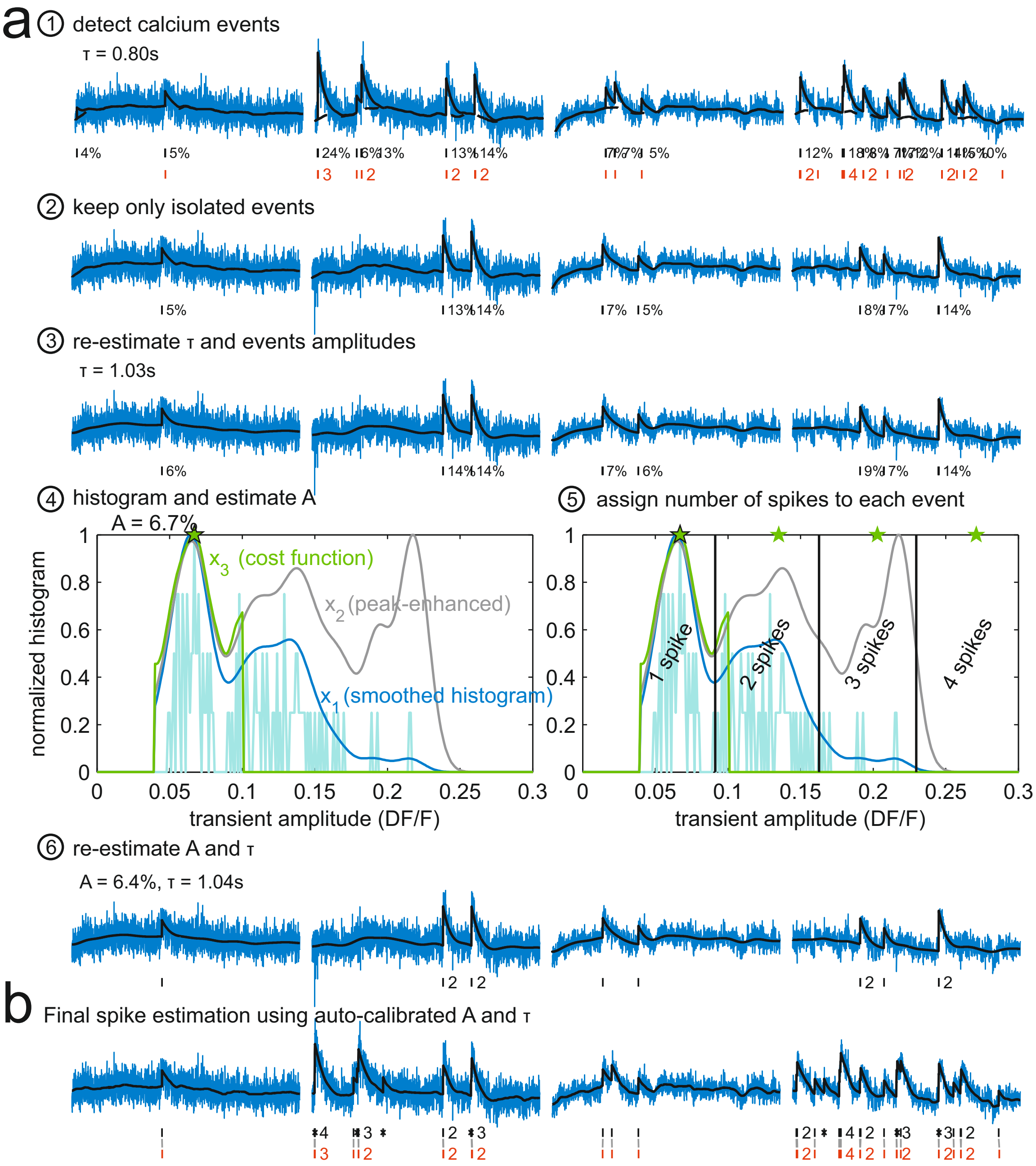

Supplementary Figure 5: **Illustration of the autocalibration algorithm on one example of real data**. Data consisted of 75 trials of 25s. **a**: The six steps of the autocalibration (for details see: *Methods*). Steps 1-2-3 are aimed at estimating the amplitudes of isolated Ca^{2+} fluorescence events. These amplitudes are indicated as percentages in black on four example trials (N.B.: electrically recorded spikes are also shown in red to compare the results to the "ground truth", but are obviously not used by the method). Steps 4-5 are aimed at assigning a number of spikes to each of these events, based on the histogram of event amplitudes (more precisely, on specific functions derived from the histogram). Step 6 finally estimates parameters A and τ. **b**: Spike estimation by MLspike, using the autocalibrated parameter.

a

			(polynomial nonlinearity modeling)		(full physiological model)			
	A (ΔF/F for 1 spike)	tau	p2	p3	saturation	Hill exponent	c0	rise time
OGB	4.90% (±1.3)	0.78s (±0.37)			0.091 (±0.06)			
GCaMP5	1.20% (±1.7)	1.63s (±0.55)	1.11 (±1.61)	0.0087 (±0.05)	2.4e-4 (±1.7e-4)	2.57 (±0.41)	1.1 (±0.73)	26.2ms (±9.6)
GCaMP6s	11.30% (±8.3)	1.87s (±0.35)	0.81 (±0.54)	-0.056 (±0.056)	7.9e-3 (±1.4e-2)	1.84 (±0.25)	0.36 (±0.64)	70.2ms (±18.7)
GCaMP6f	3.41% (±1.04)	0.76s (±0.17)	0.85 (±1.05)	-0.006 (±0.11)	7e-4 (±1.2e-3)	2.99 (±0.53)	1.5 (±0.59)	15.6ms (±7.1)

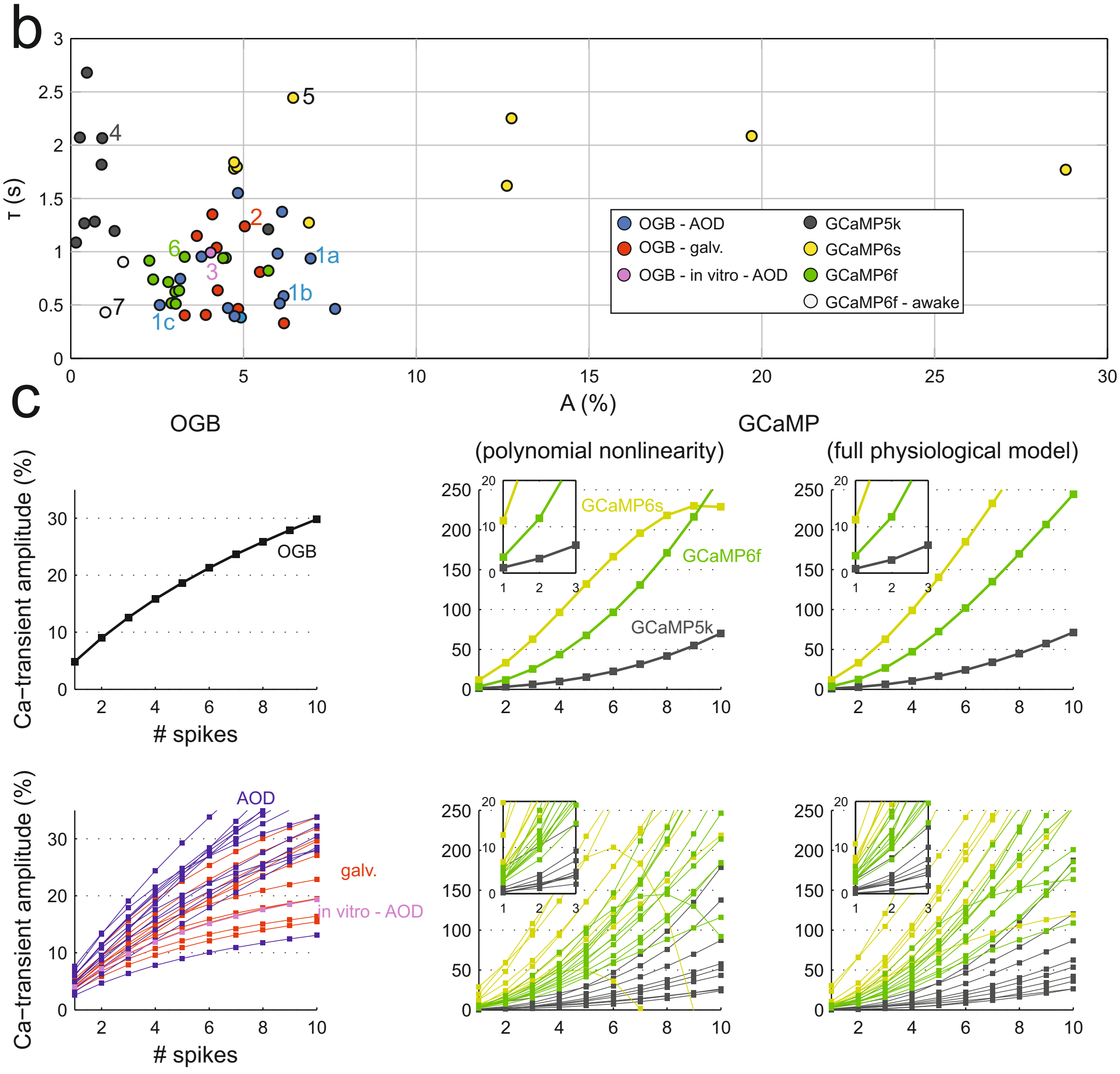

Supplementary Figure 6: **Calibration report: model parameters obtained using simultaneous imaging and electrophysiology. a**: Table summarizing "calibrated" model parameters, i.e., minimizing the discrepancy between the measured fluorescence and that predicted by the electrically recorded spikes. Our estimations of parameter A for GECIs are slightly lower than those reported in [2], due to our different normalization convention (division of the background-subtracted signal by baseline alone, rather than by (baseline-0.7xbackground)). **b**: Plotting single-exponential decay constant τ against unitary calcium transient A ("calibrated" parameter values) allows comparing the characteristics of the different indicators (see Supplementary Methods and Supplementary Fig. 9 for more details, including on different response models). Numbers near points correspond to example traces in Fig. 5a,b. **c**: Amplitude

of fluorescence transients as function of spikes recorded from cells in (a). Non-linearities in the response dynamics were accounted for by either a cubic polynomial based on heuristics [3], or by a more physiological model based on Ca^{2+} dynamics as predicted by the Hill equation[4]. In both cases, the model parameters were obtained through calibration as described above. Top: mean. Bottom: same data as (top), but plotted separately for parameters obtained from each neuron in (b). Some variability was encountered among cells. In addition, in some GCaMP6 cases the response amplitude predicted by the polynomial model re-decreased to near-zero values for large spike numbers, underscoring the limitations of this non-physiology based model.

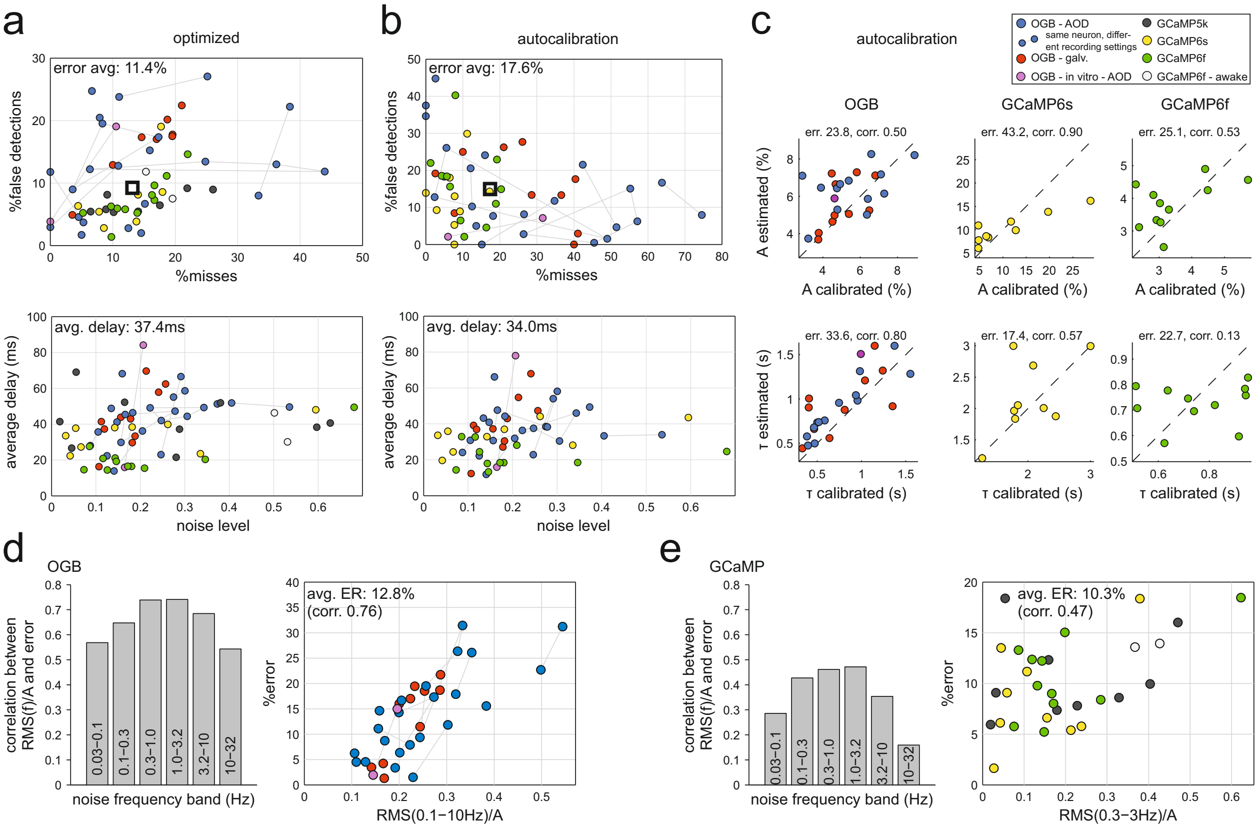

<u>Supplementary Figure 7</u>: **Model parameter estimation details; noise level investigations. a:** Optimized parameters; top: parameter estimation on our complete set of real data shows an equilibrated distribution of misses and false detections (black square marks position of average - same data and graphical conventions as in Fig. 5b,c, in all panels). Bottom: mean spike time error shown as a function of noise level. **b:** Same as (a), but for autocalibrated parameters (no electrophysiology data were used). Note that estimations using optimized parameters (a) are overall more equilibrated in terms of misses and false detections than in (b), where some points are largely imbalanced due to errors in parameter estimation by the autocalibration ((a) and (b) top are differently scaled). Average timing error, however, was the same in the two cases. **c:** Correlation between autocalibrated parameter values and those obtained by calibration using simultaneous electrophysiological recordings (see Methods). Two models were tried for the GECIs: a cubic polynomial with instantaneous risetime (left) and the "Hill exponent" (right), i.e., finite risetime and amplitude derived from the Hill equation [4]. Same color conventions as in (a). **d-e:** Correlation values between ER and noise RMS/A in various narrow

frequency bands, for synthetic Ca^{2+} indicator OGB (d) and GECIs (e). In the OGB case, a correlation peak is clearly seen around 1Hz, despite strong correlation of the noise power among different bands. The latter is due to global-effect factors such as: quality of staining, physiological condition of the preparation, photonic noise, etc. In the case of GECIs, the correlation between ER and noise level was altogether weaker: despite the small value of some of the cells' parameter A (leading to low SNR values and hampering estimations of transients evoked by single spikes), the SNR and estimation accuracy were excellent for bursts, thanks to the probe's strong supralinearity. Both for OGB and GECIs, the *precise* frequency band maximizing correlation differed slightly from the 0.1-3Hz band used throughout our study (e.g., yielding $\rho = 0.76$ *vs.* 0.69 in the case of OGB (d)). This is possibly due to small differences in the power frequency spectrum of actual *vs.* simulated unitary fluorescence transients. However, both for OGB and GECIs, correlation between ER and noise power dropped above 10Hz independently of the precise band chosen to quantify noise level (d), thus underscoring the stronger impact of low- frequency with respect to high-frequency noise on spike estimation accuracy.

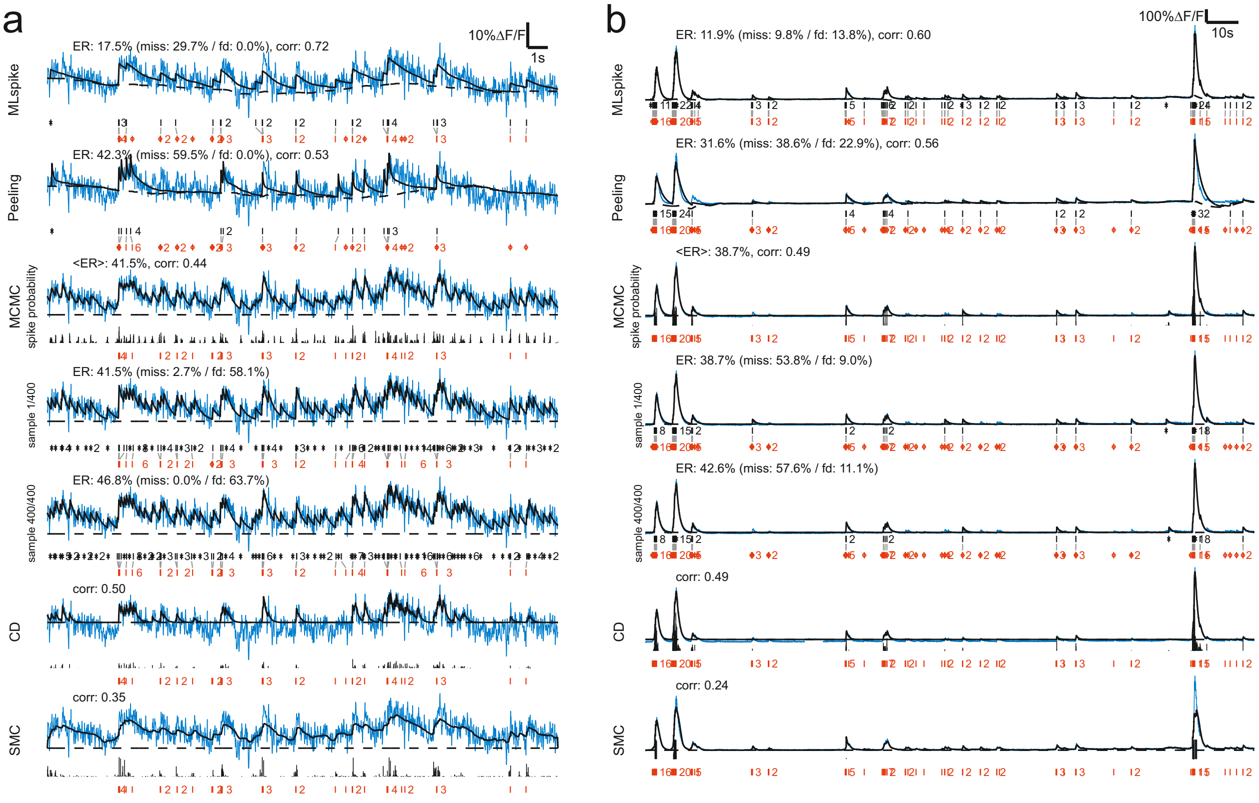

Supplementary Figure 8: **Algorithm comparison: examples.** Performance of *MLspike*, *Peeling*, *MCMC* (spike probability and two sample spike trains), *CD* and *SMC* on two examples (see legend of Fig. 6a for details), which illustrate the variety of signals that *MLspike* can handle. **a:** This OGB *in vivo* recording shows *MLspike's* better handling of several kinds of noises than the other algorithms, including slow drifts and temporally correlated faster fluctuations. In particular, these noises strongly confuse parameter estimation for the *MCMC* algorithm (*A* being underestimated, leading respectively to many false detections). **b:** Example from the GCaMP6s

dataset: although the probe's strong nonlinearities pose a strong challenge also to *MLspike*, it handles them better than the other algorithms. *Peeling* underestimates small transients (resulting in many misses) but overestimates large transients (resulting in many false positives); *MCMC* overestimates *A*, leading to many misses.

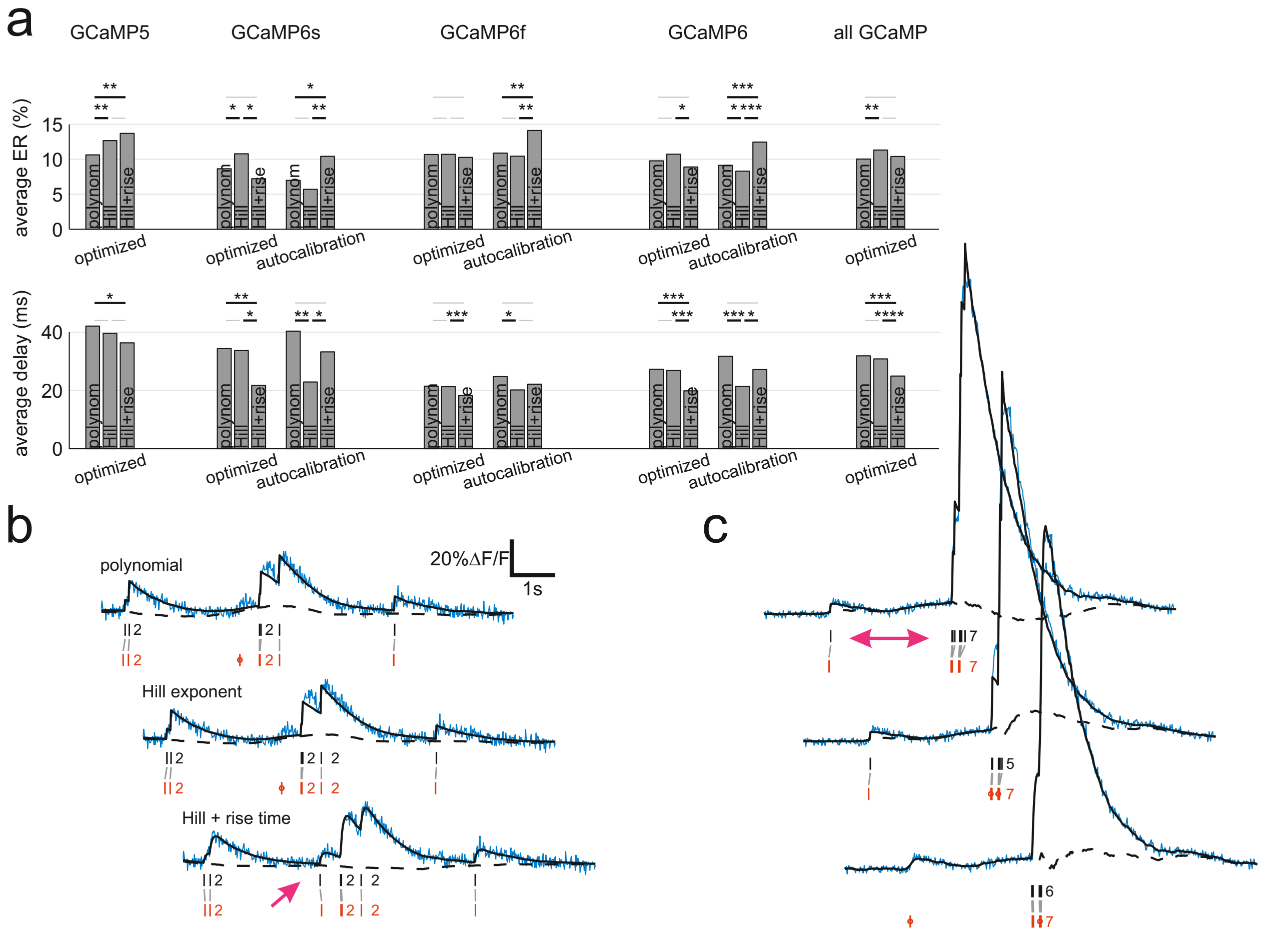

Supplementary Figure 9: **Comparing three models for the non-linearities of GECIs** Three different models were tested to account for the non-linear response dynamics of GCaMP5 and GCaMP6: (i) "Hill exponent": instantaneous risetime but amplitude derived from the Hill equation[4], (ii) "Hill + risetime": same as (i) but with finite risetime, and (iii) "polynomial": instantaneous risetime and amplitude modeled heuristically via a cubic polynomial [3] see Methods). **a:** Comparison of estimation accuracy between the three non-linearity models applied on the three different GECIs, for optimized and autocalibrated (the latter failed for GCaMP5) parameter sets. Top: spike estimation error, bottom: spike timing error (one-sided Wilcoxon signed ranked test, *: p<0.05, **: p<0.01, ***: p<0.001). The poorer performance of the finite as compared to the instantaneous risetime model in the autocalibration case might result from some cases where the true risetime was shorter than the one used in the model (which was fixed to the values specified in Supplementary Fig. 6a), thus inducing large errors in the estimation of the other model parameters. **b:** Example spike estimation using the three different models. In one case, an isolated spike is correctly detected (arrow) only by the model with finite rise time. **c:** same as (b), but during a period displaying both an isolated spike and a burst. Only the polynomial model succeeds in estimating the correct number of spikes in both cases (arrow).

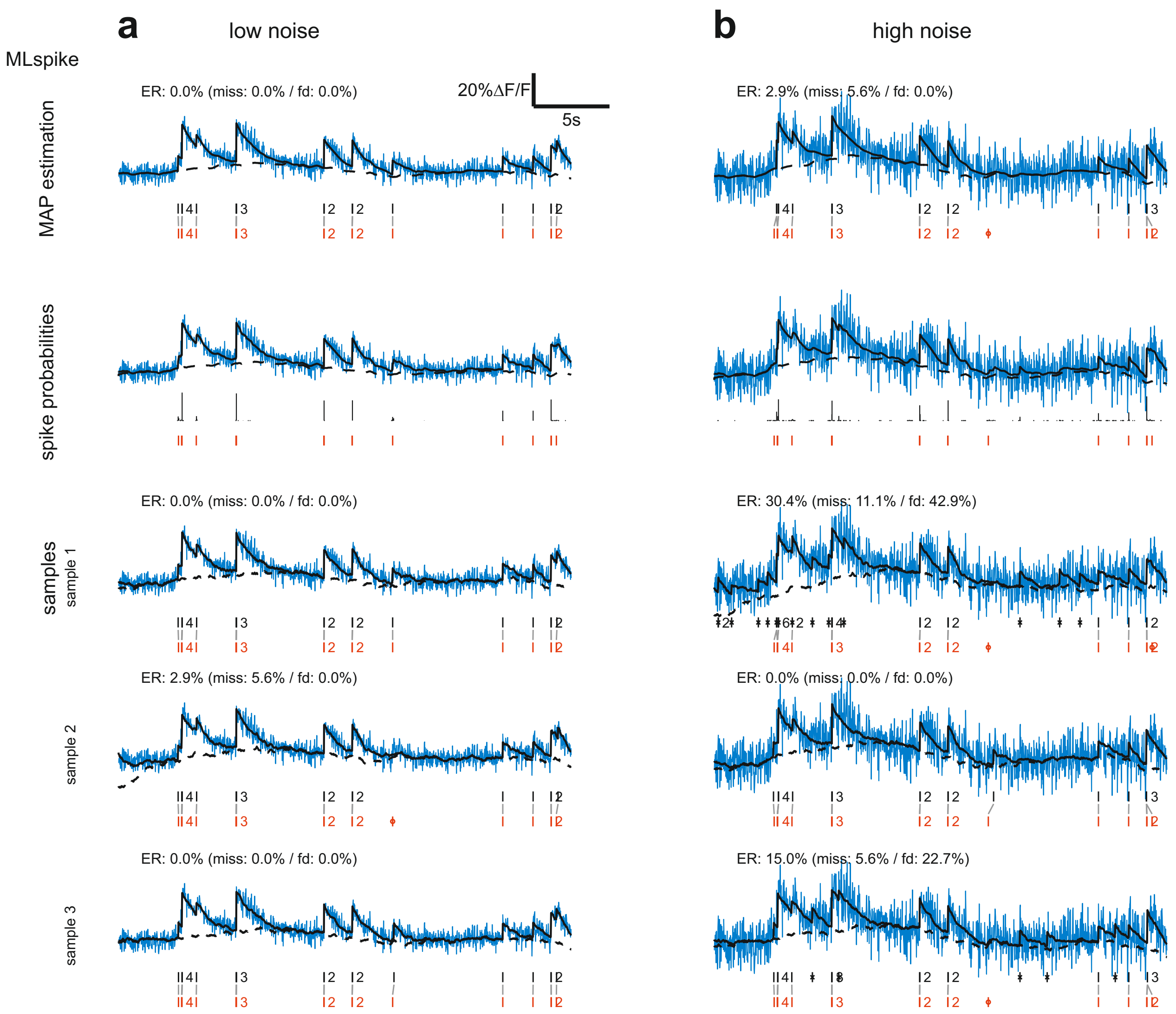

***<u>Supplementary Figure 10</u>*: *MLspike* used to return either the unique MAP estimate, spike probabilities, or sample spike trains.** The original equations of *MLspike* can be modified so as to return either spike probabilities or sample spike trains (see Methods). The latter provide additional information on the uncertainty of the estimations. Here, the three different estimations have been applied to simulated data with two different levels of noise. **a:** When noise is low, estimation uncertainty is low too: as a consequence, spiking probabilities are very focused in time, and sample estimates are very similar one to another (3 examples are shown). **b:** When noise is high, spiking probabilities are more scattered, and sample estimates display large variability. All graphical conventions are the same as in Fig. 6 and Supplementary Fig. 8.

<u>Supplementary Table 1:</u> *Summary of parameters and methods used in the different Figures of the article (including the Supplementary ones).*

Dataset		Simulations				Real data		
		flat baseline but unknown level	flat baseline, known level	drifts & fluctuations	autocalibration	optimized (OGB: 24 cells; GCaMP5k: 9; 6s: 9; 6f: 11; 6f-awake: 2)	autocalibration (OGB: 24 cells; GCaMP6s: 9; 6f: 11)	1,011 neurons (OGB)
Figures		2, S1	S2	3, S3, S4	4b,c	5a,b,d, 6, S6, S7a,d,e, S8, S9	4a, 5c,d, 6, S5, S7b,c, S8, S9a	7
Characteristics of the data	Spikes	poisson	poisson	poisson	poisson events (fix rate), but events can be bursts	physiological spikes		
	Frame rate	different frame rates	different frame rates	100Hz	100Hz	from 30Hz to 200Hz		30Hz
	real A and τ	10% - 1s			random A (4%-10%) and τ (0.4s - 1.6s)	could be estimated through calibration from electrophysiology for all cells; for GCaMP estimations, we used the polynomial nonlinearity model except in S9 that compares 3 models		could be estimated through electr. calibr. only for patched neuron
	nonlinearity parameter(s)	γ=0.1	γ=0	γ=0.1 or γ=0 (in S4)	γ=0.1			
	noise	white noise	white noise	filtered noise (3 different spectra)	slow drift + white noise	photonic noise + system noise + biological noise + calcium activity not related to spiking and possible oversimplifications of the model…		
Details of the estimations	Algorithm(s) tested	*MLspike*	*MLspike*, *Peeling*	*MLspike*, *Peeling* (S4 only)	*MLspike*	*MLspike*	*MLspike*, *Peeling*, *MCMC*, *CD*, *SMC*	
	Physiological parameters	A, τ and γ known by the algorithm			A and τ autocalibrated, γ fixed to 0.1	calibration value given to the algorithm	autocalibrated (except *Peeling* : fixed)	
	Noise and drift parameters	optimized	optimized	optimized	σ auto-calibrated, η fixed	optimized	*MLspike*: σ autocalibrated, η fixed; *Peeling*: 3 parameters fixed; others: autocalibrated	

Supplementary Note 1: “Factor Box”

This document illustrates qualitatively how the performance of MLspike depends on various primary and secondary factors.

The simulation on the right is characterized by a spiking rate of 1Hz, low photonic noise (RMS = 0.01) and small baseline drift/fluctuations. It serves as “starting point”. In the following simulations, various factors will be varied, one at a time, whereas the other simulation parameters stay unchanged (unless specified otherwise).

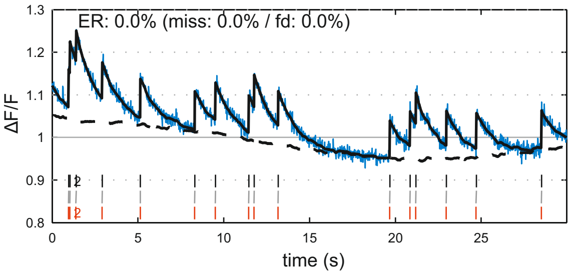

I Primary factors for MLspike estimation accuracy

1) Noise level

Increasing **photonic noise** results in poorer performance. However, it is not the high frequency part of this noise that hampers estimation. Rather, the critical part of the noise is around 1Hz, as is shown further below.

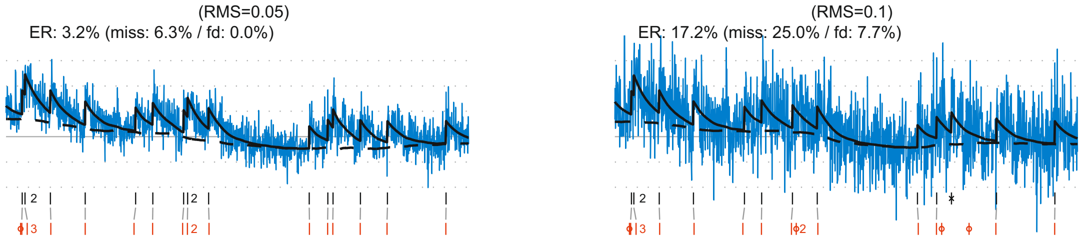

Performance is quite robust against **low frequency noises** up to ~0.1Hz.

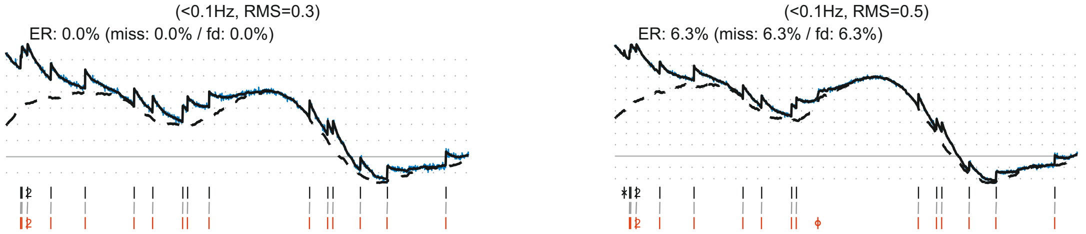

Noises in the **"worst band" (0.1-3Hz)** most strongly impact on the estimation's accuracy.

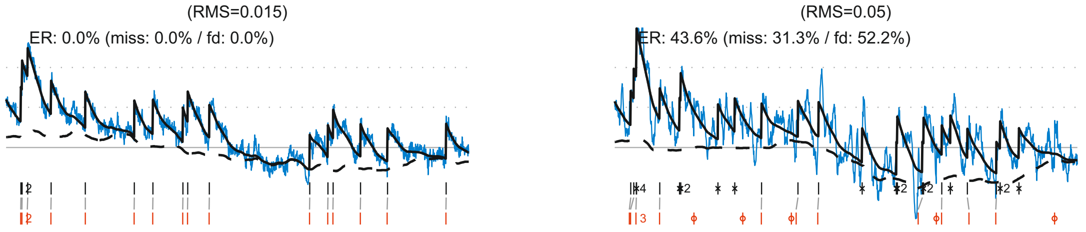

2) Spiking rate and spiking regularity

Higher **spiking rates** result in poorer estimations, mainly because it becomes more difficult to correctly estimate the baseline. Therefore, the **spiking patterns** are also important: bursts alternating with silent periods ease baseline estimation (first row), wheras near to regularly dense spiking (last row) impairs it. Note that noise also hampers baseline estimation: in the bottom-left example, baseline and spikes are exquisitely recovered but when noise hides the individual spike onsets, estimation quality rapidly deteriorates (not shown). Note also that the ability to recover fast spiking pattern depends on the **calcium sensor properties**, in particular **decay time constant** and **saturation**. The simulations here assume an OGB dye, see next section for GCaMP6 probes.

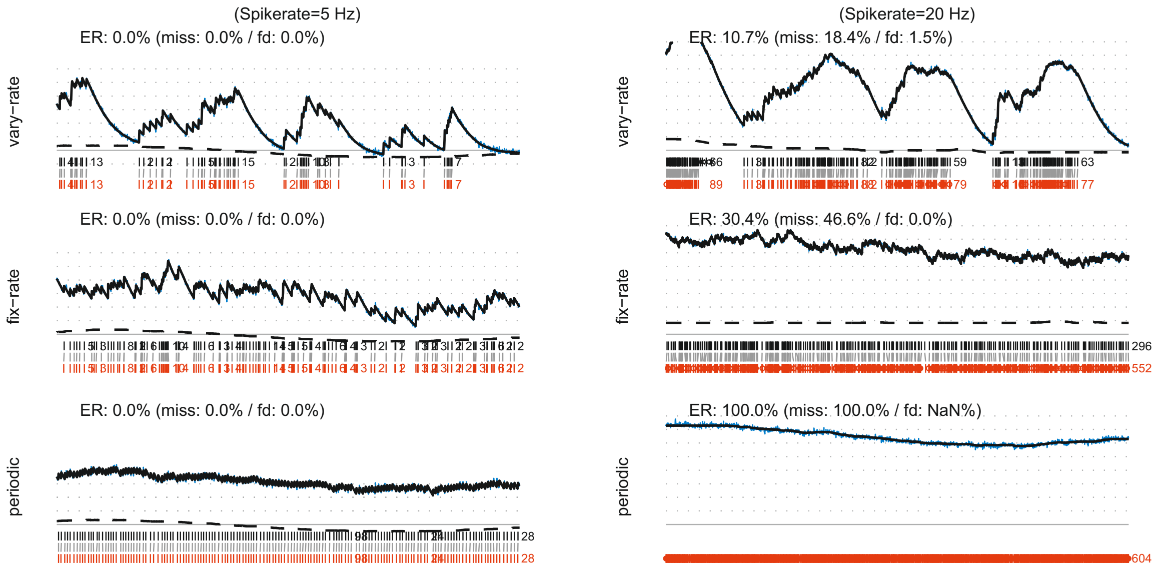

II Secondary factors for MLspike estimation accuracy

A number of factors can be considered as "secondary", in the sense that their effect can be reduced to the effect of one or more of the primary factors described above.

1) Ca-fluorescence transient amplitude

Decrease of unitary Ca transient amplitude has a similar effect as increasing noise: more errors occur, as it becomes more difficult to distinguish spikes from noise. Baseline estimation is also impaired.

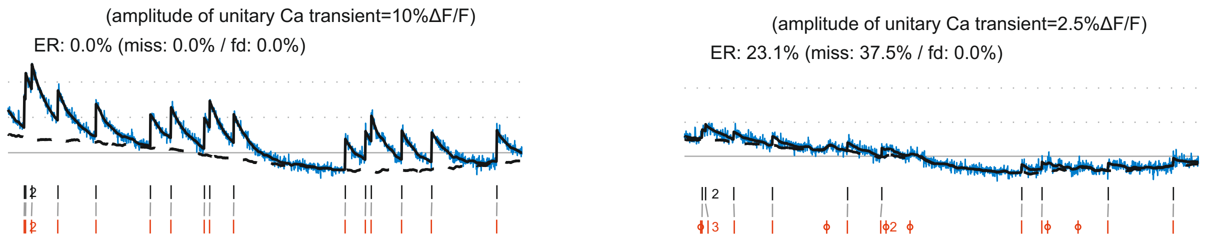

2) Laser intensity

Increasing laser intensity decreases the photonic noise in the ΔF/F signals (by the square root of the increase in signal). This improves spike estimation significantly only if the noise in the 0.1-3Hz frequency band is of photonic origin, but not otherwise.

3) Changing sampling rate at constant laser power

Left panel vs. middle panel: When decreasing sampling rate by scanning the same field of view at lower speed, the SNR in individual frames increases (by the square root of the speeds' ratio, due to increased dwell time: here, a 4x slower scanning results in 2x higher SNR). Yet, the estimation quality remains similar (main text, Fig. 2a, right), because the noise level (quantified as RMS in the 0.1-3Hz band) remains constant. However, at low noise levels, slow sampling may obviously reduce temporal precision (e.g. Supp. Fig. 1a).
Left panel vs. right panel: When decreasing sampling rate by scanning a larger field of view without changing scanning speed, the SNR in individual frames does not increase (dwell time remains unchanged), reducing estimation accuracy.

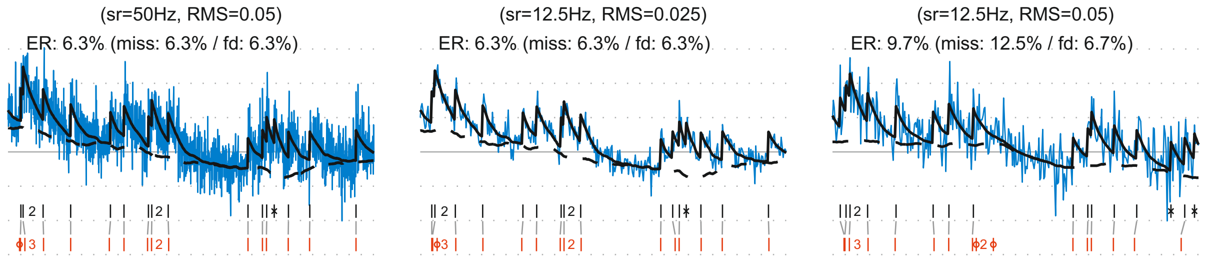

4) Contamination

The fluorescence recorded from one cell can be contaminated by the signal from another one (or from the neuropil, e.g., when imaging with low numerical aperture or from deep locations). Dealing with this kind of "noise" is particularly challenging. Nevertheless, the algorithm can handle up to more than 30% contamination, as shown below. The top and middle traces represent the contribution of the primary and of the contaminating cell to the effectively recorded signal (bottom trace).
Note that, here, keeping the unitary transient amplitude set to its "uncontaminated" value (middle column) rather than to its actual value (right column) improved the estimations.

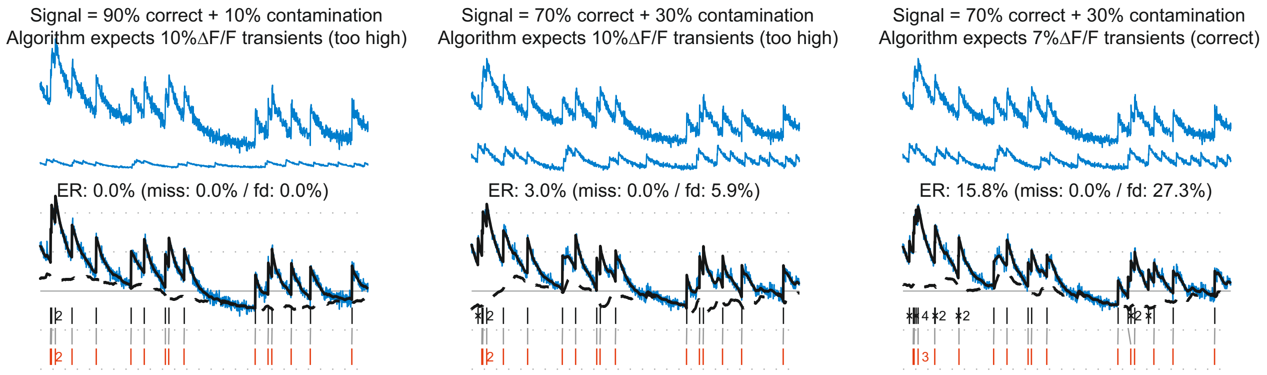

5) Numerical aperture of the objective

Lower numerical apertures result in lower SNR because of weaker signal (laser beam less focused) and in more contamination by other cells and/or the neuropil (degraded spatial resolution). The graph here displays SNR computed as unitary peak response to stimulation / standard deviation of the fluorescence baseline.
Data stems from whole-cell patched GCaMP6f neurons in mice cortical slices recorded with a variable NA next to the objective's back aperture. Objective: Olympus XLUMPLFLN 20x, NA=1.0.

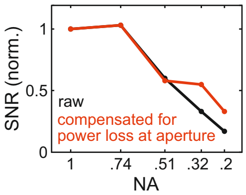

6) Recording depth

The effects of recording depth can vary importantly between different experimental conditions and depend on **tissue transparency**, the efficacy of **scattered fluorescence collection**, and the locality and sparseness of the **Ca probe's distribution**. In general, recording deep inside scattering tissues reduces the signal (less ballistic excitation photons reach the sample), thus demanding high laser power, and increases contamination by other cells and/or the neuropil. This contamination originates both from the neighborhood of the recorded cell (due to a larger point spread function of the focused beam), and from the superficial layers where the probe is excited despite a non-focused laser beam, because the latter is strong and only weakly attenuated by tissue scattering (see simulations below). In order to avoid contamination from surface fluorescence upon deep imaging, it is thus preferable to load only the imaged area with the calcium sensor, rather than the full volume.

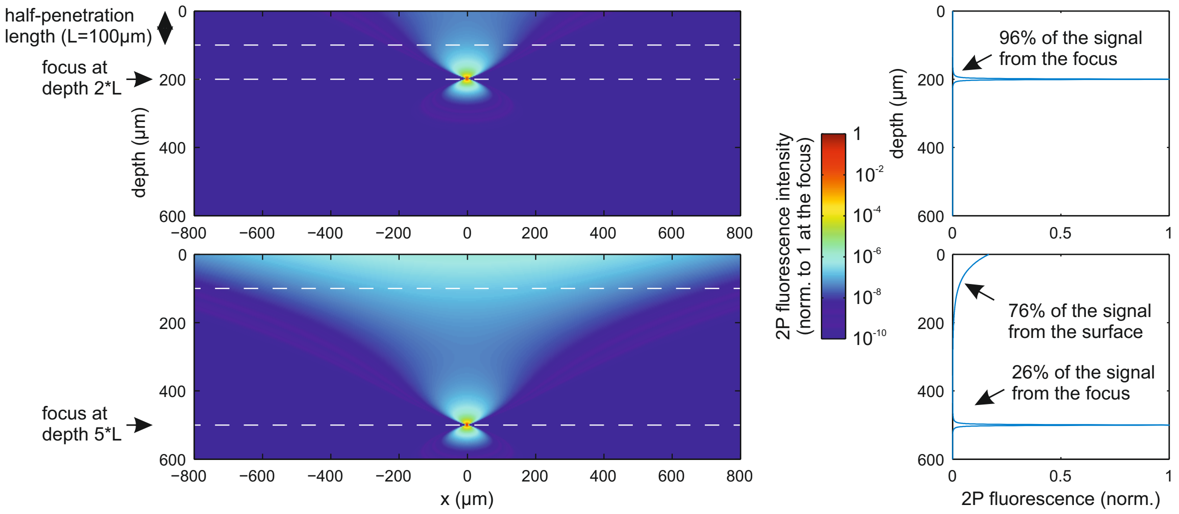

7) Calcium sensors

Carefully exploring all factors making some sensors preferable over others in general (ability to do chronic imaging, target specific cells, pharmacological side-effects, etc.) goes beyond the scope of this work. Here, we characterize only those that directly affect the ability to estimate spikes from calcium recordings. Their **transient amplitude** for one spike directly influences the SNR; their **rise** and **decay times** influence the temporal precision of the estimations and the ability to follow high spiking rates – which can also be limited by **saturation**; finally, the a priori **knowledge** on the exact values of these parameters and their **variability** also influence the ability to perform autocalibration: e.g., we still miss knowledge on the exact function governing nonlinearities of GCaMP6 sensors.
In the graphs below we compare spike estimation accuracy on data simulated using characteristics of GCaMP6s and GCaMP6f. At low spiking rate (left column), GCaMP6s clearly outperforms GCaMP6f thanks to its larger unitary fluorescence transients: it can accomodate a noise 5 times larger at comparable level of accuracy (note that OGB dye would be positioned halfway). This advantage is progressively lost at higher spiking rates (top left). Finally, only GCaMP6f can follow (Poisson-statistics) trains of spikes at 20sp/s (bottom right), while GCaMP6s is limited to about 5sp/s.

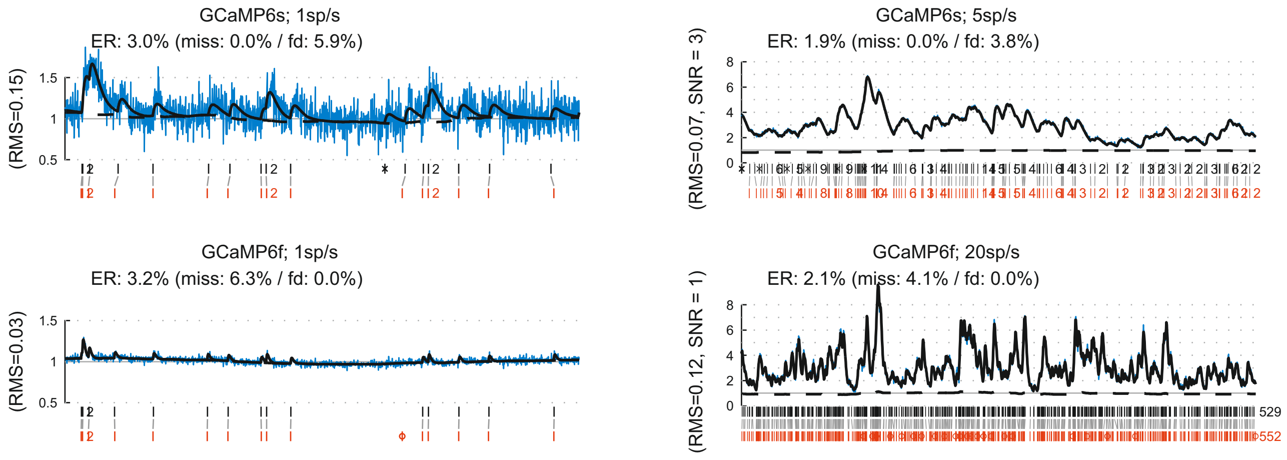

III Fine-tuning of MLspike algorithm

MLspike estimation accuracy obviously depends on its parameter settings. Here, we assume physiological parameters (A, tau, nonlinearity of the probe) to be known (see next section on autocalibration when this is not the case) and investigate the effects of the three remaining parameters: *sigma, drift*, and *spikerate*.
These parameters code for the a priori level of expected photonic noise, slow drifts and spiking activity present in the data. Their relative settings therefore influence how the algorithm interprets ambiguous variations of the signal.

1) A priori white noise level: parameter *sigma*

sigma is the expected RMS of a temporally uncorrelated noise. When the signals are unambiguous, because the noise is small (first line: RMS=0.01, 2 spikes/s), a wide range of *sigma* values leads to correct estimations. However, when the noise is large (second line: RMS=0.04), low values of *sigma* amount to "over-trusting the data " and cause false detections (as well as an underestimation of the baseline level:, left). Conversely, high *sigma* values amount to "not trusting the data enough", increasing the number of misses (right).

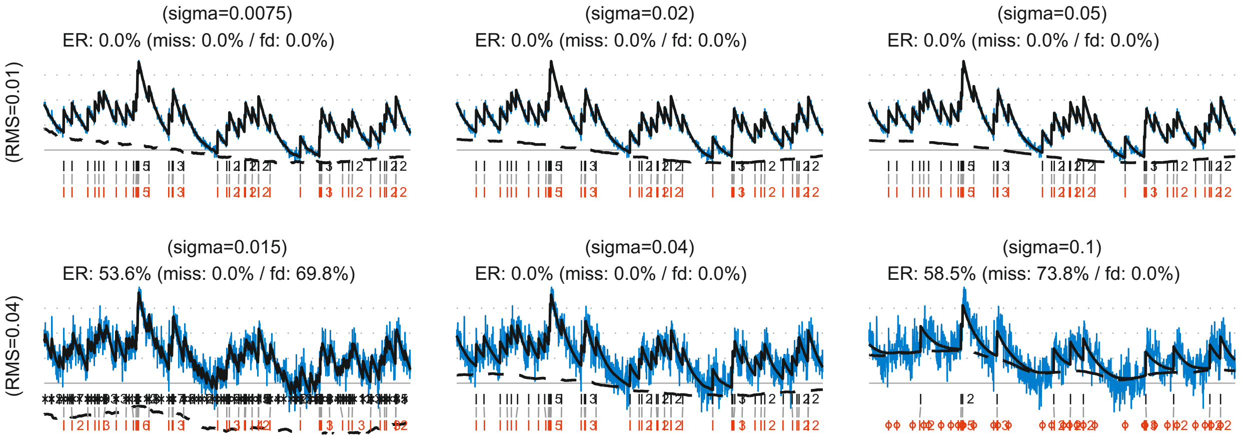

Parameter *sigma* can be **autocalibrated** from the data themselves. The simulations below show estimated sigma values and resulting spike estimations for the same signals as above (left and center). Autocalibration is possible also when the noise is temporally correlated (right).

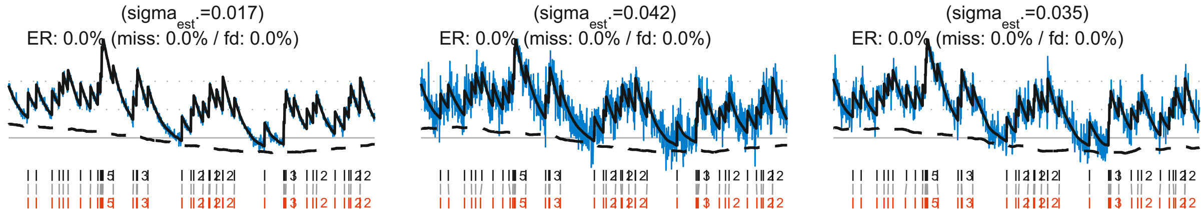

2) A priori drift level: *drift* parameter

The *drift* parameter has a similar effect as *sigma*, but for low-frequency noises: Setting a low value results in fluctuations to be mistaken for spikes (second line, left), while setting a high value results in spikes to be mistaken for drifts/fluctuations (right). However, estimations are robust with respect to mis-setting the drift parameter (first line), provided the signals are not too ambiguous (second line).

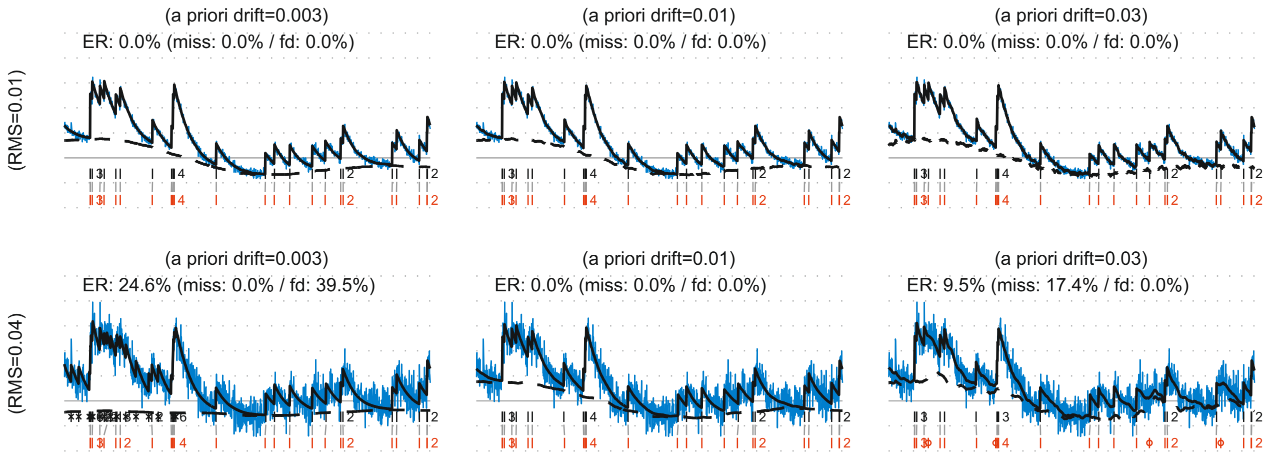

3) A priori spiking activity level: parameter *spikerate*

Increasing parameter *spikerate* increases the algorithm's tendency to assign spikes (i.e. decreases misses but increases false detections). Note that the optimal value for *spikerate* is not necessarily the true spike rate. Note also that the 3 parameters *sigma, drift* and *spikerate* together control only 2 degrees of freedom of the estimation, as increasing one of them has exactly the same effect as decreasing the other two. In practice, parameter *spikerate* can thus be assigned a fix value (we usually use 1sp/s) while the other two parameters are fine tuned if necessary.

IV Autocalibration of physiological parameters

Parameters *A* (the fluorescence transient amplitude for one spike) and *τ* (decay time constant) can be autocalibrated. The autocalibration algorithm (see Fig. 4a and Sup. Figs. 5) detects isolated calcium events and uses a histogram of all event amplitudes in order to assign a number of spikes to each event and to finally return estimated values for *A* and *τ*.

1) Noise level

The same factors that affect MLspike's estimations affect also the autocalibration. Below we show that, e.g., different types of noises affect autocalibration differently: slow drifts (second row) have only little effect, as opposed to "worst band" (0.1-3Hz) noises, which affects autocalibration critically.

For each estimation, purple arrows indicate the events detected by the autocalibration, their amplitude (ΔF/F in %) and the number of spikes they were assigned. Larger noise can lead to less events to be detected or to falsely detected ones, to approximate amplitude estimations and to wrong spike number assignments. Eventually this results in an erroneous estimation of A (see the indicated goodness of estimation expressed as ratios A_{est}/A and $τ_{est}/τ$), and in spike estimations errors.

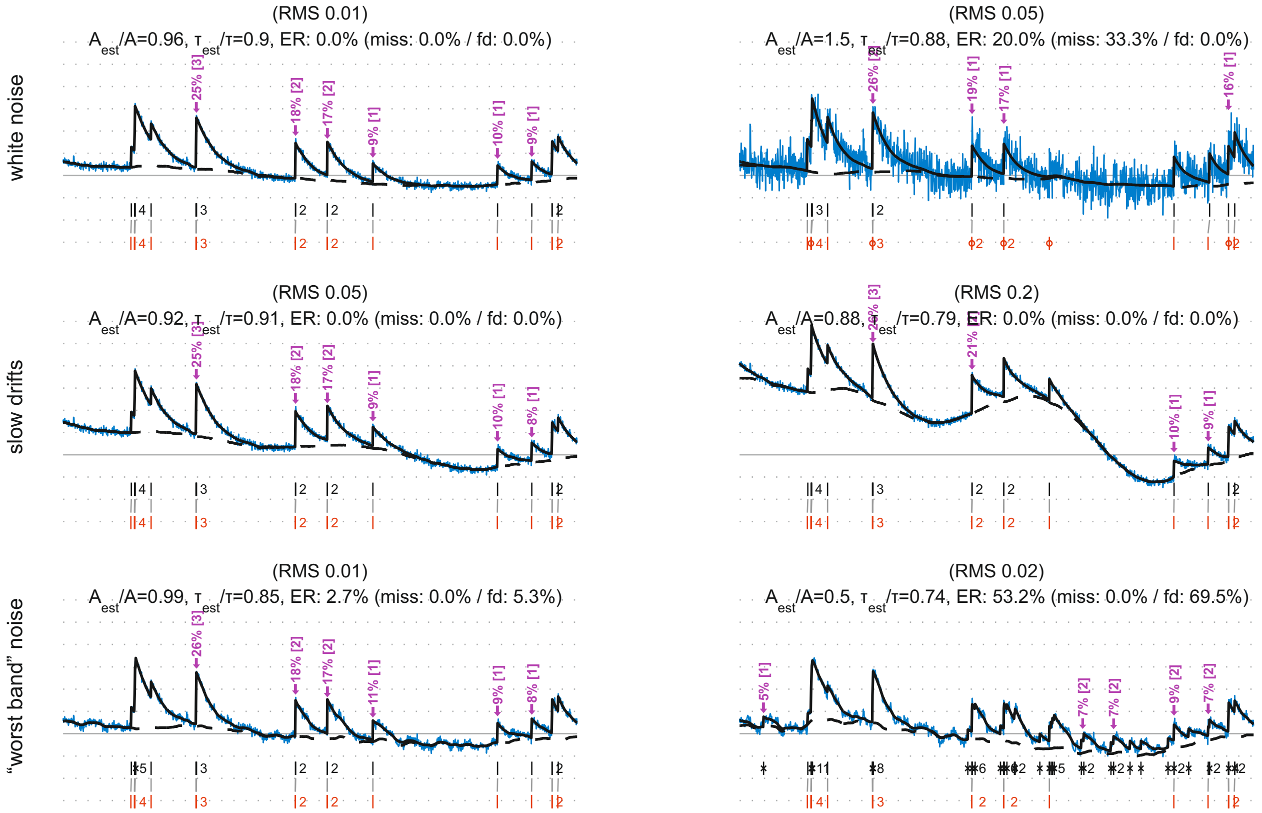

2) Data length

Autocalibration requires a sufficient number of isolated spikes. Autocalibration on longer data therefore yields more accurate results than on short ones, provided the quality of the data remains constant in time (see below results for T=20s on the left vs. T=60s on the right, at an average spiking rate of 1sp/s).

For the same reason autocalibration becomes less accurate at higher spiking rates (see Fig. 4b), as less isolated events can be detected.

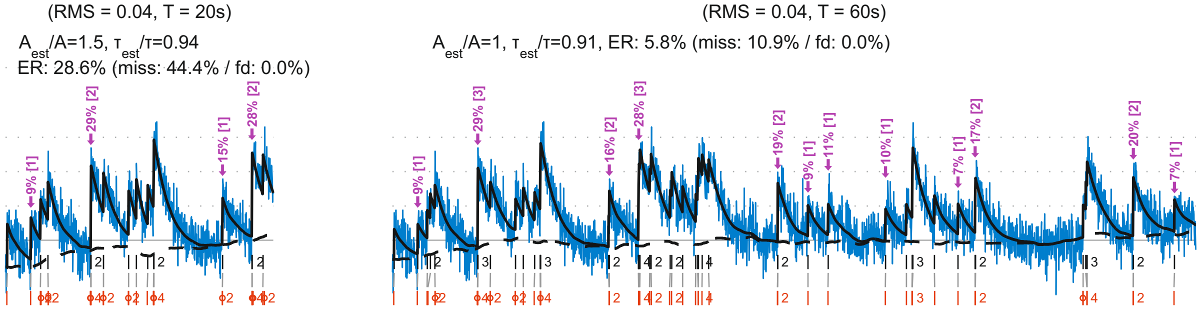

Supplementary References

1. Lütcke, H., Gerhard, F., Zenke, F., Gerstner, W. & Helmchen, F. Inference of neuronal network spike dynamics and topology from calcium imaging data. *Frontiers in Neural Circuits* **7,** 201 (2013).
2. Chen, T.-W. *et al.* Ultrasensitive fluorescent proteins for imaging neuronal activity. *Nature* **499,** 295–300 (2013).
3. Akerboom, J. *et al.* Optimization of a GCaMP calcium indicator for neural activity imaging. *J. Neurosci.* **32,** 13819–13840 (2012).
4. Helmchen, F. in *Handbook of Neural Activity Measurement* (eds. Brette, R. & Desthexhe, A.) 362–409 (2012).
