# Supplementary figures and images for: Accurate spike estimation from noisy calcium signals for ultrafast three-dimensional imaging of large neuronal populations in vivo
Source: Nat Commun. 2016 Jul 19;7:12190. doi: 10.1038/ncomms12190 (PMC4960309; doi:10.1038/ncomms12190)

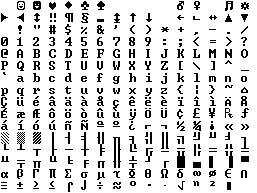

Supplement: Supplementary Software — MATLAB implementation of the MLspike and autocalibration algorithms are provided here. Eventual software upgrades will be available at https://github.com/MLspike [file ncomms12190-s3.zip › MLspikes-160606/brick/ascii8x12.png]

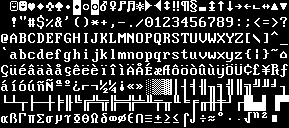

Supplement: Supplementary Software — MATLAB implementation of the MLspike and autocalibration algorithms are provided here. Eventual software upgrades will be available at https://github.com/MLspike [file ncomms12190-s3.zip › MLspikes-160606/brick/ascii8x16.png]
