# Supplementary material for: Accurate spike estimation from noisy calcium signals for ultrafast three-dimensional imaging of large neuronal populations in vivo
Source: Nat Commun. 2016 Jul 19;7:12190. doi: 10.1038/ncomms12190 (PMC4960309; doi:10.1038/ncomms12190)
Supplement: Supplementary Software — MATLAB implementation of the MLspike and autocalibration algorithms are provided here. Eventual software upgrades will be available at https://github.com/MLspike [file ncomms12190-s3.zip › MLspikes-160606/spikes/html/spk_demo.html]

Autocalibration demo 

# Autocalibration demo

This script demonstrates on simulated data how to use the MLspike and autocalibration algorithms. To see how to run the algorithms, go directly to section 'Spike estimation'.

## Contents

- Generate spikes
- Random A and tau, and random level of noise
- Generate calcium
- Spike estimation (using naive parameters)
- Auto-calibration
- Spike estimation (using autocalibrated parameters)

## Generate spikes

We first generate some simulated data. Spike trains are generated using spk\_gentrain function (the data consists of 6 trials of 30s).

Note the usefull function spk\_display to display both spikes and calcium time courses.

```
% parameters
ntrial = 6;
T = 30;
rate = 1;

% generate spikes
spikes = spk_gentrain(rate,T,'bursty','repeat',ntrial);

% display
figure(1)
spk_display([],spikes,[])
```

## Random A and tau, and random level of noise

We draw randomly the parameters governing the calcium dynamics A (DF/F amplitude for one spike) and tau (decay time constant), as well as the level of noise in the data, sigma.

```
amin = .04;
amax = .1;
a = amin * exp(rand(1)*log(amax/amin)) % log-uniform distribution

taumin = .4;
taumax = 1.6;
tau = taumin * exp(rand(1)*log(taumax/taumin))

sigmamin = .005;
sigmamax = .05;
sigma = sigmamin * exp(rand(1)*log(sigmamax/sigmamin))
```

```
a =

    0.0983


tau =

    0.9311


sigma =

    0.0387
```

## Generate calcium

We generate calcium signals corresponding to the above spike trains using these parameters. Note that some additional parameters (OGB dye saturation and drift parameter) are fixed.

```
% parameters
dt = .02; % 50Hz acquisition
pcal = spk_calcium('par');
pcal.dt = dt;
pcal.a = a;
pcal.tau = tau;
pcal.saturation = .1; % saturation level is fixed
pcal.sigma = sigma; % noise level
pcal.drift.parameter = [5 .015]; % drift level (#harmonics and amplitude of them)

% generate calcium
calcium = spk_calcium(spikes,pcal);

% display
figure(1)
spk_display(dt,spikes,calcium)
drawnow
```

## Spike estimation (using naive parameters)

We first apply the MLspike algorithm using some fixed parameters. To run the algorithm on other data, set variable 'calcium' as a vector time courses of the calcium signal if there is only one trial, or to a cell array of such time courses if there are many trials.

```
% parameters
% (get the default parameters)
par = tps_mlspikes('par');
% (indicate the frame duration of the data)
par.dt = dt;
% (set physiological parameters)
par.a = 0.07; % DF/F for one spike
par.tau = 1; % decay time constant (second)
par.saturation = 0.1; % OGB dye saturation
% (set noise parameters)
par.finetune.sigma = .02; % a priori level of noise (if par.finetune.sigma
                          % is left empty, MLspike has a low-level routine
                          % to try estimating it from the data
par.drift.parameter = .01; % if par.drift parameter is not set, the
                           % algorithm assumes that the baseline remains
                           % flat; it is also possible to tell the
                           % algorithm the value of the baseline by setting
                           % par.F0
% (do not display graph summary)
par.dographsummary = false;

% spike estimation
[spikest fit drift] = spk_est(calcium,par);

% display
figure(1)
spk_display(dt,{spikes spikest},{calcium fit drift})
set(1,'numbertitle','off','name','MLspike alone')
```

```
tps_mlspike 6/6
```

The above graphs compare spikes estimated using autocalibration followed by MLspike (in black) with the original spikes (in red), as well as the estimated drift (dashed black) and fit to data. Because the parameters A, tau and sigma used for the estimation might not be appropriate, the estimation might not be very successful.

## Auto-calibration

Now we want to estimate the original spikes from the calcium signals rather than using fixed parameter. First we run the autocalibration algorithm to attempt estimating, from the calcium signals directly, the values of parameters A, tau and sigma.

```
% parameters
% (get default parameters and set frame duration)
pcal = spk_autocalibration('par');
pcal.dt = dt;
% (set limits for A and tau)
pcal.amin = amin;
pcal.amax = amax;
pcal.taumin = taumin;
pcal.taumax = taumax;
% (set saturation parameter)
pcal.saturation = 0.1;
% (give real values to the algorithm for display purposes - they obviously won't be used in the estimation!)
pcal.realspikes = spikes;
pcal.reala = a;
pcal.realtau = tau;
% (when running MLspike from spk_autocalibratio, do not display graph summary)
pcal.mlspikepar.dographsummary = false;

% perform auto-calibration
[tauest aest sigmaest] = spk_autocalibration(calcium,pcal)
```

```
estimate noise level
detect events
sub-select events
estimate tau
histogram and assign number of spikes to events
re-estimate A and tau

tauest =

    0.8597


aest =

    0.0947


sigmaest =

    0.0254
```

The above graphs illustrate some steps of the performed autocalibration. As can be seen, estimated values for A and tau are pretty close to the real values used for the simulation. The estimated value for sigma is generally lower that the original sigma: this is normal, as the MLspike generally performs best when it uses a value for sigma slightly lower than the real one (using the real one tends to generate more misses).

## Spike estimation (using autocalibrated parameters)

After this autocalibration we can apply the MLspike algorithm by using the autocalibrated parameters.

```
% parameters
par = tps_mlspikes('par');
par.dt = dt;
% (use autocalibrated parameters)
par.a = aest;
par.tau = tauest;
par.finetune.sigma = sigmaest;
% (the OGB saturation and drift parameters are fixed)
par.saturation = 0.1;
par.drift.parameter = .01;
% (do not display graph summary)
par.dographsummary = false;

% spike estimation
[spikest fit drift] = spk_est(calcium,par);

% display
figure(2)
spk_display(dt,{spikes spikest},{calcium fit drift})
set(2,'numbertitle','off','name','Auto-calibration + MLspike')
```

```
tps_mlspike 6/6
```

Spikes estimated in such a way now match the original spikes with a lower error rate.

Published with MATLAB® R2013a
