# Supplementary material for: Accurate spike estimation from noisy calcium signals for ultrafast three-dimensional imaging of large neuronal populations in vivo
Source: Nat Commun. 2016 Jul 19;7:12190. doi: 10.1038/ncomms12190 (PMC4960309; doi:10.1038/ncomms12190)
Supplement: Supplementary Software — MATLAB implementation of the MLspike and autocalibration algorithms are provided here. Eventual software upgrades will be available at https://github.com/MLspike [file ncomms12190-s3.zip › MLspikes-160606/spikes/html/spk_factorbox.html]

spk\_factorbox 

## Contents

- FACTOR BOX
- 0 Starting point: "standard" simulation
- I Effect of primary factors on MLspike estimation accuracy
- I/1) Noise level
- I/2) Spiking rate and spiking regularity
- II Effect of secondary factors
- II/1) Ca-fluorescence transient amplitude
- II/2) increasing laser intensity
- II/3) changing sampling rate at constant laser power
- II/4) contamination
- II/5) numerical aperture
- II/6) recording depth
- II/7) calcium sensors
- III Fine-tuning of MLspike algorithm
- III/1) A priori noise level: parameter 'sigma'
- III/1.1) Automatic estimation of parameter 'sigma'
- III/2) A priori drift level: parameter 'drift'
- III/3) A priori spiking rate: parameter 'spikerate'
- IV Autocalibration of physiological parameters
- IV/1) Noise level
- IV/1.1) Photonic noise
- IV/1.2) Low frequency noises (0.001Hz - 0.1 Hz)
- IV/1.3)
- IV/2) Data length

## FACTOR BOX

This document illustrates qualitatively how the performance of MLspike depends on various primary and secondary factors.

The simulation on the right is characterized by a spiking rate of 1Hz, low photonic noise (RMS = 0.01) and small baseline drift/fluctuations. It serves as “starting point”. In the following simulations, various factors will be varied, one at a time, whereas the other simulation parameters stay unchanged (unless specified otherwise).

## 0 Starting point: "standard" simulation

The simulation we start from is characterized by low levels of photonic noise and drift-fluctuations and a spiking rate of 1Hz

display

## I Effect of primary factors on MLspike estimation accuracy

## I/1) Noise level

Increasing **photonic noise** results in poorer performance. However, it is not the high frequency part of this noise that hampers estimation. Rather, the critical part of the noise is around 1Hz, as shown further below.

Performance is quite robust against **low frequency noises** up to ~0.1Hz (this advantage is somewhat lost at spiking rates above ~10Hz).

Noises in the **"worst band" (0.1-3Hz)** most strongly impact on the estimation's accuracy.

## I/2) Spiking rate and spiking regularity

Higher spiking rates result in poorer estimations, mainly because it becomes more difficult to correctly estimating the baseline. Therefore, the spiking pattern is also important: bursts alternating with silent periods eases baseline estimation (first row), wheras near to regularly dense spiking (last row) impairs it. Note that noise also hampers baseline estimation: in the bottom-left example, baseline and spikes are exquisitely recovered but when noise hides the individual spike onsets, estimation quality rapidly deteriorates.

## II Effect of secondary factors

A number of factors can be considered as "secondary", in the sense that their effect can be reduced to the effect of one or more of the primary factors described above.

## II/1) Ca-fluorescence transient amplitude

Decrease of unitary Ca transient amplitude (fed to the algorithm) has a similar effect as increasing noise: more misses occur, as it becomes more difficult to distinguish spikes from noise. Baseline estimation is also impaired.

## II/2) increasing laser intensity

Increasing laser intensity decreases the photonic noise in the ?F/F signals (by the square root of the increase in signal). This improves spike estimation significantly only if the noise in the 0.1-3Hz frequency band is of photonic origin, but not otherwise.

## II/3) changing sampling rate at constant laser power

Left vs. middle panel: When decreasing sampling rate by scanning the same field of view at lower speed, the SNR in individual frames increases (by the square root of the speeds' ratio, due to increased dwell time: here, a 4x slower scanning results in 2x higher SNR). Yet, the estimation quality remains similar (main text, Fig. 1c, right), because the noise level (quantified as RMS in the 0.1-3Hz frequency band) remains constant. However, at low noise levels, slow sampling may obviously reduce temporal precision (Supp. Fig. 1). Left panel vs. right panel: When decreasing sampling rate by scanning a larger field of view without changing scanning speed, the SNR in individual frames does not increase (dwell time remains unchanged), reducing estimation accuracy.

## II/4) contamination

The fluorescence recorded from one cell can be contaminated by the signal from another one (or by the signal from the neuropil, as can happen for example when imaging with low numerical aperture or from deep locations). Dealing with this kind of "noise" is particularly challenging. Nevertheless, the algorithm handles quite well up to more than 30% contamination, as shown below. Keeping the amplitude set to its "uncontaminated" rather than to its actual value further penalizes the contaminating Ca transients leading to improved estimations (middle vs. right panel).

## II/5) numerical aperture

Lower numerical apertures result in lower SNR, because of more photonic noise (black) and more contamination by other cells and/or the neuropil (red). SNR was computed as: unitary peak response amplitude/ devided by the standard deviation of the fluorescence baseline. SNR was then normalized dividing bythe value at maximal NA. Data stem from whole-cell patched GCaMP6f neurons in mice cortical slices recorded with a variable NA. Points and errorbars represent, respectively, mean and std of 10 unitary fluorescence transients.

## II/6) recording depth

The effects of recording depth can vary importantly between different experimental conditions and depend on on tissue transparency, the efficacy of scattered fluorescence collection, and the locality and sparseness of the Ca probe’s distribution. In general, recording deep inside scattering tissues reduces the signal (less ballistic excitation photons reach the sample), thus demanding high laser power, and increases contamination by other cells and/or the neuropil. This contamination originates both from the neighborhood of the recorded cell (due to a larger point spread function of the focused beam), and from the superficial layers where the probe is excited despite a non-focused laser beam, because the latter is strong and only weakly attenuated by tissue scattering (see simulations below). In order to avoid contamination from surface fluorescence upon deep imaging, it is thus preferable to load only the imaged area with the calcium sensor, rather than the full volume.

```
26% of the signal at the focus
```

## II/7) calcium sensors

Carefully exploring all factors making some sensors preferable over others in general (ability to do chronic imaging, target specific cells, pharmacological side-effects, etc.) goes beyond the scope of this work. Here, we characterize only those that directly affect the ability to estimate spikes from calcium recordings. Their transient amplitude for one spike directly influences the SNR; their rise and decay times influence the temporal precision of the estimations and the ability to follow high spiking rates – which can also be limited by saturation; finally, the a priori knowledge on the exact values of these parameters and their variability also influence the ability to perform autocalibration: e.g., we still miss knowledge on the exact function governing nonlinearities of GCaMP6 sensors.

In the graphs below we compare spike estimation accuracy on data simulated using characteristics of GCaMP6s and GCaMP6f. At low spiking rate (left column), GCaMP6s clearly outperforms GCaMP6f thanks to its larger unitary fluorescence transients: it can accomodate a noise 5 times larger at comparable level of accuracy (note that OGB dye would be positioned halfway). This advantage is progressively lost at higher spiking rates (top left). Finally, only GCaMP6f can follow (Poisson-statistics) trains of spikes at 20sp/s (bottom right), while GCaMP6s is limited to about 5sp/s.

## III Fine-tuning of MLspike algorithm

MLspike estimation accuracy obviously depends on its parameter settings. Here, we assume physiological parameters (A, tau, nonlinearity of the probe) to be known (see next section on autocalibration when this is not the case) and investigate the effects of the three remaining parameters: sigma, drift, and spikerate.

These parameters code for the a priori level of expected photonic noise, slow drifts and spiking activity present in the data. Their relative settings therefore influence how the algorithm interprets ambiguous variations of the signal.

## III/1) A priori noise level: parameter 'sigma'

sigma is the expected RMS of a temporally uncorrelated noise. When the signals are unambiguous, because the noise is small (first line: RMS=0.01, 2 spikes/s), a wide range of sigma values leads to correct estimations. However, when the noise is large (second line: RMS=0.04), low values of sigma amount to "over-trusting the data " and cause false detections (as well as an underestimation of the baseline level:, left). Conversely, high sigma values amount to "not trusting the data enough", increasing the number of misses (right).

## III/1.1) Automatic estimation of parameter 'sigma'

Parameter sigma can be autocalibrated from the data themselves. The simulations below show estimated sigma values and resulting spike estimations for the same signals as above (left and center). Autocalibration is possible also when the noise is temporally correlated (right).

## III/2) A priori drift level: parameter 'drift'

The drift parameter has a similar effect as sigma, but for low-frequency noises: Setting a low value results in fluctuations to be mistaken for spikes (second line, left), while setting a high value results in spikes to be mistaken for drifts/fluctuations (right). However, estimations are robust with respect to mis-setting the drift parameter (first line), provided the signals are not too ambiguous (second line).

## III/3) A priori spiking rate: parameter 'spikerate'

Increasing parameter spikerate increases the algorithm’s tendency to assign spikes (i.e. decrease misses but increase false detections). Note that the optimal value for spikerate is not necessarily the true spike rate. Note also that the 3 parameters sigma, drift and spikerate together control only 2 degrees of freedom of the estimation, as increasing one of them has exactly the same effect as decreasing the other two. In practice, parameter spikerate can thus be assigned a fix value (we usually use 1sp/s) while the other two parameters are fine tuned if necessary.

## IV Autocalibration of physiological parameters

Parameters A (the fluorescence transient amplitude for one spike) and ? (decay time constant) can be autocalibrated. The autocalibration algorithm (see Fig. 1e,f and Sup. Figs. 7,8) detects isolated calcium events and uses a histogram of all event amplitudes in order to assign a number of spikes to each event and to finally return estimated values for A and ?.

## IV/1) Noise level

The same factors that affect MLspike’s estimations affect also the autocalibration. Below we show that, e.g., different types of noises affect autocalibration differently: slow drifts (second row) have only little effect, as opposed to “worst band” (0.1-3Hz) noises, which affects autocalibration critically.. For each estimation, purple arrows indicate the events detected by the autocalibration, their amplitude (?F/F in %) and the number of spikes they were assigned. Larger noise can lead to less events to be detected or to falsely detected ones, to approximate amplitude estimations and to wrong spike number assignments. Eventually this results in an erroneous estimation of A (see the indicated goodness of estimation expressed as ratios Aest/A and ?est/?), and in spike estimations errors.

## IV/1.1) Photonic noise

## IV/1.2) Low frequency noises (0.001Hz - 0.1 Hz)

## IV/1.3)

## IV/2) Data length

For the same reason autocalibration becomes less accurate at higher spiking rates (see Fig. 1f), as less isolated events can be detected.

```
seed 20
T=20s -> A_{est}/A=1.5, \tau_{est}/\tau=0.94, ER: 28.6% (miss: 44.4% / fd: 0.0%)
T=60s -> A_{est}/A=0.89, \tau_{est}/\tau=0.88, ER: 0.9% (miss: 1.8% / fd: 0.0%)
```

Published with MATLAB® R2013a
